# Phylogeographic patterns and conservation implications of the endangered Chinese giant salamander

**DOI:** 10.1002/ece3.5014

**Published:** 2019-03-07

**Authors:** Zhi‐Qiang Liang, Wei‐Tao Chen, Deng‐Qiang Wang, Shu‐Huan Zhang, Chong‐Rui Wang, Shun‐Ping He, Yuan‐An Wu, Ping He, Jiang Xie, Chuan‐Wu Li, Juha Merilä, Qi‐Wei Wei

**Affiliations:** ^1^ Key Laboratory of Freshwater Biodiversity Conservation, Ministry of Agriculture of China, Yangtze River Fisheries Research Institute Chinese Academy of Fishery Sciences Wuhan China; ^2^ Hunan Fisheries Science Institute Changsha China; ^3^ Collaborative Innovation Center for Efficient and Health Production of Fisheries in Hunan Province Changde China; ^4^ College of Life Science Southwest University Chongqing China; ^5^ The Key Laboratory of Aquatic Biodiversity and Conservation of Chinese Academy of Sciences, Institute of Hydrobiology Chinese Academy of Sciences Wuhan China; ^6^ Pearl River Fisheries Research Institute Chinese Academy of Fishery Science Guangzhou China; ^7^ Administrative Office of The National Giant Salamander Nature Reserve of Zhangjiajie Hunan China; ^8^ Ecological Genetics Research Unit, Research Programme in Organismal and Evolutionary Biology, Faculty of Biological and Environmental Sciences, Department of Biosciences University of Helsinki Helsinki Finland

**Keywords:** *Andrias davidianus*, conservation, genetic diversity, geographic partition, phylogeography

## Abstract

Understanding genetic diversity patterns of endangered species is an important premise for biodiversity conservation. The critically endangered salamander *Andrias davidianus*, endemic to central and southern mainland in China, has suffered from sharp range and population size declines over the past three decades. However, the levels and patterns of genetic diversity of *A. davidianus* populations in wild remain poorly understood. Herein, we explore the levels and phylogeographic patterns of genetic diversity of wild‐caught *A. davidianus* using larvae and adult collection with the aid of sequence variation in (a) the mitochondrial DNA (mtDNA) fragments (*n* = 320 individuals; 33 localities), (b) 19 whole mtDNA genomes, and (c) nuclear recombinase activating gene 2 (*RAG2*; *n* = 88 individuals; 19 localities). Phylogenetic analyses based on mtDNA datasets uncovered seven divergent mitochondrial clades (A–G), which likely originated in association with the uplifting of mountains during the Late Miocene, specific habitat requirements, barriers including mountains and drainages and lower dispersal ability. The distributions of clades were geographic partitioned and confined in neighboring regions. Furthermore, we discovered some mountains, rivers, and provinces harbored more than one clades. *RAG2* analyses revealed no obvious geographic patterns among the five alleles detected. Our study depicts a relatively intact distribution map of *A. davidianus* clades in natural species range and provides important knowledge that can be used to improve monitoring programs and develop a conservation strategy for this critically endangered organism.

## 1 INTRODUCTION

**Figure 1:**
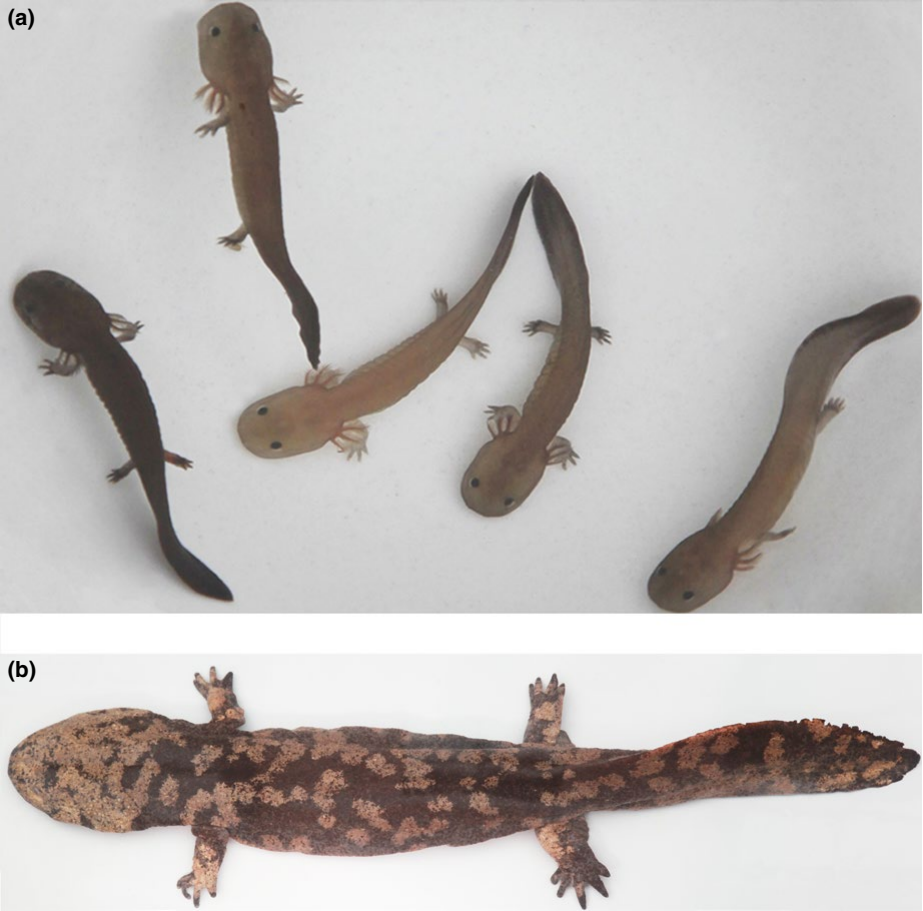
Wild‐caught larvae (a) and an adult (b) of *A. davidianus* from Zhangjiajie, Hunan Province. Photograph: Zhiqiang Liang

The Chinese giant salamander (*Andrias davidianus*) (Figure 1) is the largest extant amphibian species in the world, and it is entirely aquatic and endemic to the montane areas of central and southern China at approximately 23.5–35°N and 100–120°E (Fu, 1993). The Chinese giant salamander is listed in CITES Appendix I as a specially protected animal (category II). Consequently, it is protected under Chinese conservation laws (Dai, Wang, & Liang, 2009) and has been assessed as Critically Endangered on the IUCN Red List (Liang, Geng, & Zhao, 2004). However, the loss of suitable habitat and human consumption of these animals has caused the range and population size of *A. davidianus* declining sharply over the past three decades (Dai et al., 2009). In order to counteract this, protection and restoration its breeding habitats, as well as release of farmed individuals to wild, have been implemented in many provinces in China since 1972 (Dai et al., 2009). However, artificial breeding and releasing programs may have translocated *A. davidianus* from unknown sources to non‐native habitats, which might have led to genetic admixture and pose risks to native populations. Thus, understanding the current patterns of genetic diversity of this critically endangered species in wild might help in formulating future management strategies and policies. In particular, identification of unique genetic lineages that are unlikely to have been subject to human‐assisted introgression could allow prioritizing populations of special conservation value.

Number of earlier studies have focused on genetic variation and levels of population differentiation in *A. davidianus* (Murphy, Fu, Upton, De Lema, & Zhao, 2000; Tao, Wang, & Zheng, 2006; Tao, Wang, Zheng, & Fang, 2005; Yan et al., 2018; Yang et al., 2011). For instance, Murphy et al. (2000) investigated genetic divergence among six *A. davidianus* populations using isozyme electrophoresis and mitochondrial DNA (mtDNA) sequences and found some genetic divergence among the analyzed populations. In addition, they detected genetic signatures of human‐assisted translocations on patterns of *A. davidianus* population differentiation. Tao et al. (2005) employed mtDNA d‐loop sequences and found significant population genetic differentiation between the Pearl River and the Yangtze River, as well as between the Pearl River and the Yellow River, whereas they did not detect any pronounced differentiation among the Pearl River populations or among the Yangtze River populations. Subsequently, Tao et al. (2006) employed mtDNA cytochrome *b* gene sequences and discovered low degree of population genetic differentiation between the Pearl River and the Yangtze River, as well as between the Yangtze River and the Yellow River. However, high level of genetic differentiation was observed between the Pearl River and the Yellow River. Using AFLP makers, Yang et al. (2011) confirmed that *A. davidianus* populations showed high genetic diversity and had dispersed from north to south. More recently, Yan et al. (2018) found that 70 wild‐caught *A. davidianus* individuals from 14 localities once harbor at least five distinctive clades based on 23,159 SNPs (single‐nucleotide polymorphism) and mtDNA markers. Furthermore, they exemplified broad genetic mixing among dinstinct clades based on mtDNA and microsatellite data for more than 1,000 farm‐bred individuals. Nevertheless, the relatively small sample sizes (especially wild‐caught specimens) and restricted geographic coverage of these studies limit the inference that can be drawn from them (Murphy et al., 2000; Tao et al., 2006, 2005; Yan et al., 2018; Yang et al., 2011). For instance, Yan et al. (2018) uncovered seven clades using a large number of farm‐bred samples, but they missed two clades when using the small sample sizes of wild‐caught individuals. A comprehensive genetic analysis using a larger sample size of wild‐caught specimens covering a more of the species extensive range is hence warranted.

**Figure 2:**
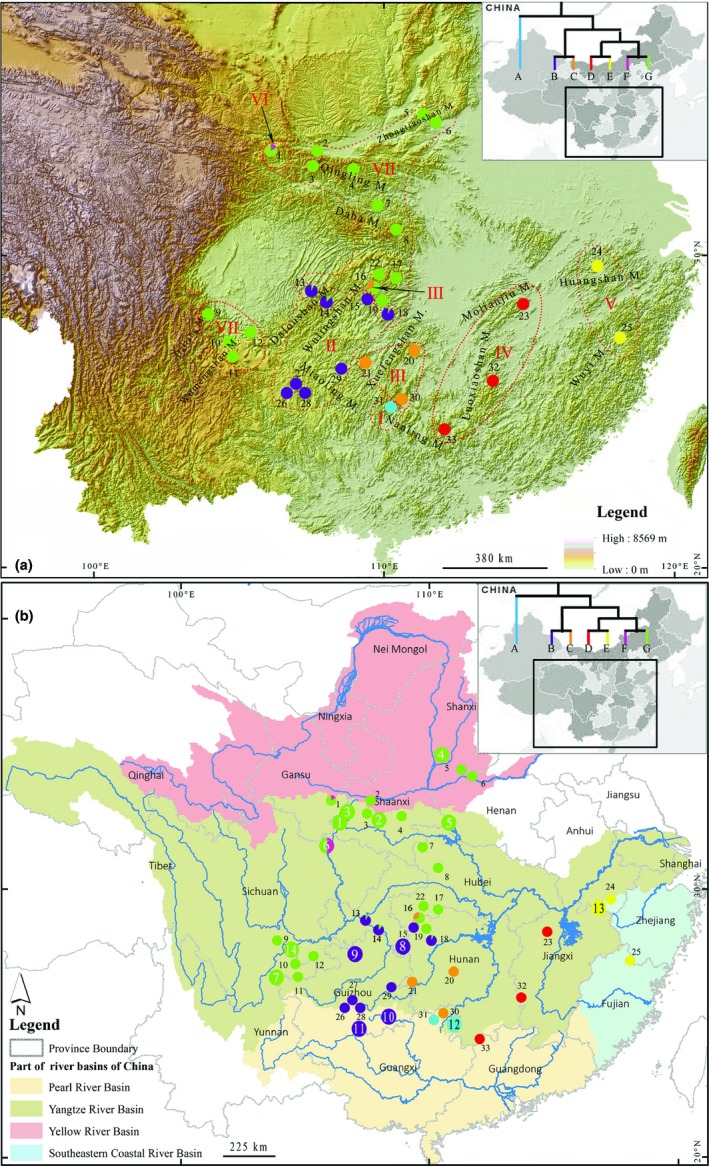
Map showing the sampling locations of *Andrias davidianus* based on mountains (a) and drainages (b). Localities are detailed in Supporting Information Table S1 in Appendix S1, and populations are presented as pie‐diagrams with slice‐size proportional to the frequency of different clades. Inset in upper right corner shows the simplified maternal genealogy with clades A–G. Colors of pie‐diagrams and tree correspond to the clades in Figure 3. Pie‐diagrams with number represent wild‐caught clades observed in Yan et al. (2018). Roman numerals indicate geographic partition of clades A–G

Amphibians are poor dispersers and sensitive to environmental changes and therefore regarded as ideal models to study historical phylogeography and local adaptation (Beebee, 2005; Zeisset & Beebee, 2008). Geological events have been identified as main factors influencing the genetic structuring of species. Events such as the orogenesis of the Qinghai–Tibetan Plateau and the Rocky Mountain system resulted in vicariance and habitat fragmentation reduced gene flow and increased genetic divergence and even led to speciation in many taxa (Antonelli, Nylander, Persson, & Sanmartin, 2009; Che et al., 2010; DeChaine & Martin, 2004; Zhou et al., 2012). The uplifting of the Qinghai–Tibetan Plateau has played a role in shaping the topography and landforms in East Asia (An, Kutzbach, Prell, & Porter, 2001; Cui, Gao, Liu, Pan, & Chen, 1996; Li, Fang, Pan, Zhao, & Song, 2001; Zheng, Powell, An, Zhou, & Dong, 2000). Central and southern China, which spans the region from the eastern Qinghai–Tibetan Plateau to the Pacific Ocean, harbors many mountain ranges (e.g., the Qinling, Wuyi and Nanling Mountains; Figure 2a). In addition to the complex drainage systems (e.g., the Yangtze River, Yellow River, Pearl River, and several coastal rivers; Figure 2b), these regions raise high species diversity for Amphibians (Zhang, 1999). Thus, these regions represent an ideal system for investigating that how these environment factors drive the genetic diversity and diversification in amphibians.

In this study, we characterized phylogeographic pattern and differentiation of *A. davidianus* on the basis of mitochondrial DNA (mtDNA) and nuclear DNA (nuDNA) utilizing a large number of wild‐caught animals sampled from area covering most of its native distribution range (http://maps.iucnredlist.org). Larvae and adult individuals were included in this study. The main aims were (a) to assess how many distinct genetic clades the *A. davidianus* consist of in the natural species ranges and where they occur, (b) to explore what factors have shaped the patterns of genetic variability and differentiation in *A. davidianus*, and (c) to offer recommendations for *A. davidianus *conservation.

## 2 METHODS AND MATERIALS

### 2.1 Sample collection and molecular data processing

The methods involving animals in this study adhered to the Laboratory Animal Management Principles of China. All experimental protocols were approved by the Ethics Committee of the Institute of Hydrobiology, Chinese Academy of Sciences.

A total of 320 wild‐caught samples were collected between May 2005 and March 2015 from 33 localities covering the most of its native distribution range of *A. davidianus* (Figure 2 and Supporting Information Table S1 in Appendix S1), which was different from the samples used in the study by Yan et al. (2018). About nineteen natural breeding caves of *A. davidianus *were reported in four provinces in China during the last twenty years (Liang et al., 2016; Luo, 2009; Luo, Liu, Zhang, Chen, & Kang, 2009; Su, Yu, & Ma, 2009; Wang, Zhang, Huang, & Fang, 2010; Wang, 2006; Xiao et al., 2014), and thirteen caves were found in Zhangjiajie in Hunan Province, China. *A. davidianus *larvae could outflow with water force from their cave outlets in winter (December to February next year) (Liang, 2015; Su et al., 2009; Wang et al., 2010). We investigated larvae in natural breeding caves from 2012 to 2017 and discovered that larvae were chance upon in ten caves during last ten years. In addition, we have found three newly breeding caves (Supporting Information Table S2 in Appendix S1). We collected 109 samples from ten natural breeding caves in our study (Supporting Information Table S2 in Appendix S1). Since 2012, we have conducted a four‐year survey to investigate the situation of the larvae outflowed from the caves in Zhangjiajie with the aid of local management agencies (Liang, 2015). We found the period of larva outleting from caves in Zhangjiajie is basically stable every year; for example, larvae began to flow out from Yuanzi cave and Wumuyu cave intermittently from early December and early January, respectively, and this phenomenon of each cave lasted for about 20 days every year (Liang, 2015). The number of larvae flowed out from one of the five caves ranged from 17 to 1,920 per year (Liang et al., 2016). Skin color was close to honey color (Supporting Information Figure S1b in Appendix S1) when larvae left from the caves for the first time, and turned into black gray (Supporting Information Figure S1c in Appendix S1) under the dim light within 12 hr (Liang, 2015). The skin of local wild adults also has speckle characteristics (Supporting Information Figure S1d,e in Appendix S1) that are stable like human fingerprints (Liang, 2015). With permission of Zhangjiajie management authorities, 54 larva individuals (Supporting Information Table S1 in Appendix S1; Figure S1a in Appendix S1) were captured and sampled in the field around water outlet of four known natural breeding caves in Zhangjiajie from 5th December to 28th January next year between 2013 and 2016. Animals were released after tissue collection at the sample sites. In total, 266 adult individuals were sampled from 29 localities. We sampled 198 adults in governmental rescue agencies. Fifty‐five of 266 adults grew up from the larvae from six caves (Supporting Information Table S2 in Appendix S1). All 320 wild‐caught samples were clear about their source and sure to come from local rivers, streams, or mountain brooks. The wild‐caught place was confirmed on the spot by sample providers and local villagers. No captive individual had been previously released in the 33 sampling localities. All sampled individuals have the same representative speckle characteristics on the skin as local wild adults.

Oral mucosa cells or exfoliated skin cuticles were obtained from the majority of specimens, and caudal fin clips were also collected from a few individuals. Animals were released after tissue collection at the site of capture. All extracted tissues were immediately stored in 99.5% alcohol at −20°C for DNA extraction.

**Figure 3:**
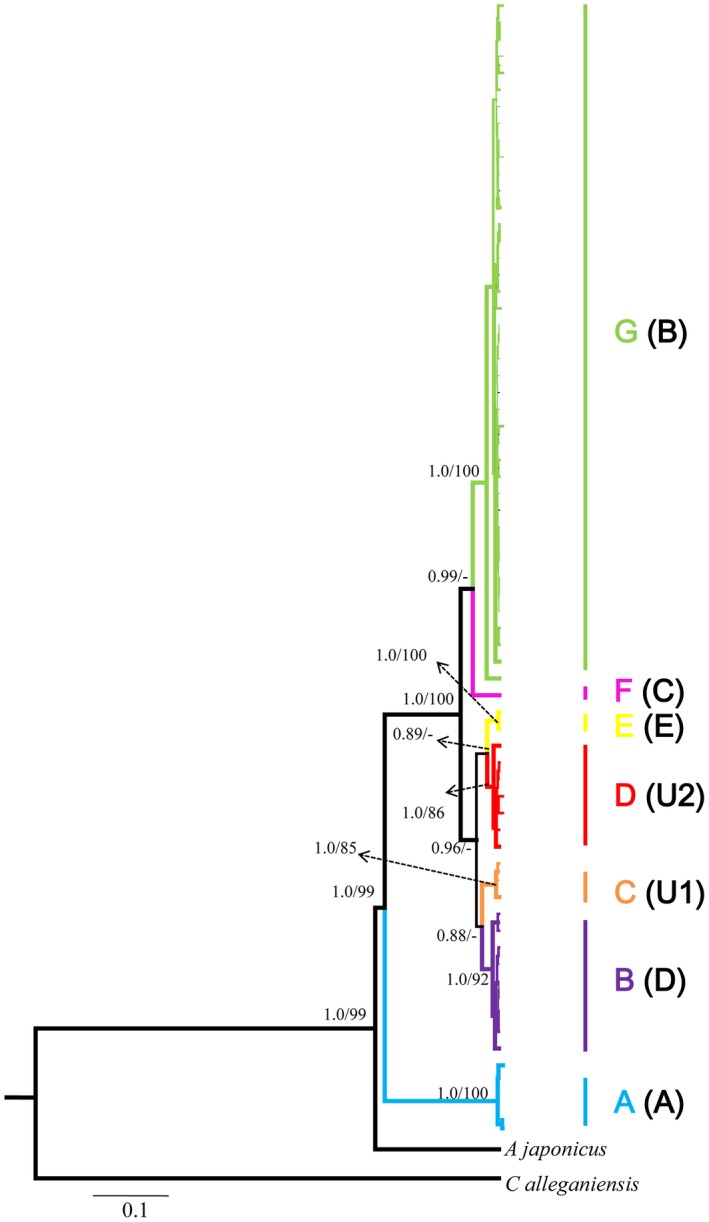
Bayesian tree based on *CCR* sequences for *Andrias davidianus*. Numbers near branches indicate Bayesian posterior probabilities and bootstrap proportions from Bayesian inferences and maximum likelihood analysis, respectively. Letters highlighted in bracket are corresponding clades in Yan et al. (2018)

The DNA was extracted using the DNA Preparation Kit (mBio, USA) according to the manufacturers instructions. The total DNA from the supernatant was purified using an Easy‐DNA Kit (Omega Bio‐Tek, Doraville, CA, USA). A pair of primers (L14764 and H16062, the 18th in Supporting Information Table S3 in Appendix S1) was designed to amplify a DNA fragment across from the partial mitochondrial cytochrome *b* gene (Cytb) of 3′‐end to the control region (*CCR*) for all individuals. Furthermore, 19 complete mtDNA genomes were amplified from a subset of samples representing each *CCR* clade to obtain robust phylogenetic trees (see Figure 3). Nineteen novel primer pairs (Supporting Information Table S3 in Appendix S1) were designed for amplifying and sequencing the complete mtDNA genomes. A published mtDNA genome of *A. davidianus* (GenBank no: NC_004926) was added to our analyses. Partial sequences from the nuclear recombinase activating gene 2 (*RAG2*; 772 bp) were also sequenced from a subset of samples representing each clade and most of the *CCR* haplotypes. A pair of primers (*RAG2*‐F and *RAG2*‐R, the 20th in Supporting Information Table S3 in Appendix S1) was designed for amplifying and sequencing the *RAG2*. A total of 88 individuals and 19 populations were included in the *RAG2 *amplification (1–11 specimens per population, mean = 4.6). All amplifications were performed in 50 μl volume with an initial denaturation period of 3 min at 94°C, which was followed by 35 cycles of 94°C for 45 s, primer‐specific annealing temperatures of 52–55°C for 1 min, 72°C for 1 min, and a final extension of 72°C for 10 min. A negative control with no template DNA was included in each PCR run. The PCR products were purified and sequenced with the same primers. DNA sequencing was performed on an ABI3730 with an ABIPRISM BigDye Terminator Cycle Sequencing Ready Reaction Kit (PerkinElmer Biosystems, Foster City, CA, USA).

### 2.2 Sequence analyses

The *CCR* sequences (998–1,361 bp) were initially edited using the DNASTAR multiple package (DNASTAR. Inc., Madison, WI, USA), aligned using MUSCLE (Edgar, 2004), and then optimized by eye in MEGA version 6.0 (Tamura, Stecher, Peterson, Filipski, & Kumar, 2013). Haplotype sequences were collapsed using DnaSP 5.10 (Librado & Rozas, 2009) based on the gaps/missing sites. *CCR* sequences provided a data matrix of 1,574 bp after alignment with two outgroups (*Andrias japonicus *and *Cryptobranchus alleganiensis*) and produced 68 ingroup haplotypes. For the 19 whole mtDNA genomes (16,311–16,503 bp), we extracted 22 transfer RNAs (tRNA), 13 protein‐coding genes with *ND6* adjusted to present the same reading direction as the other genes, and 2 ribosomal RNA genes (rRNA) from the genomes by eye. These 37 genes were combined to produce 15,684 bp concatenated sequences, which were aligned along with those of the two outgroup species.

Nuclear gene sequences containing more than one ambiguous site were resolved using PHASE 2.1.1 (Stephens, Smith, & Donnelly, 2001), accepting the results with a probability >90%. The input files for PHASE were generated using SEQPHASE (Flot, 2010). Recombination tests for detecting the longest nonrecombining region for the nuclear locus were conducted using the online version of IMGC (http://hammerlab.biosci.arizona.edu/IMGC/IMGC.html; Woerner, Cox, & Hammer, 2007) using the default settings. Identical haplotypes for phased nuDNA alleles were collapsed using DnaSP 5.10. All newly obtained sequences were deposited in GenBank (Supporting Information Table S1 in Appendix S1).

### 2.3 Phylogenetic analyses

Two other species of Cryptobranchidae, *Andrias japonicus* (GenBank: AB208679) and *Cryptobranchus alleganiensis* (GenBank: GQ368662), were selected as outgroups because of their close relationships with *A. davidianus*. The phylogenetic relationships among mitochondrial haplotypes were reconstructed using Bayesian inference (BI) and maximum likelihood (ML) for the *CCR* sequences and mtDNA genome, respectively. We employed the best‐fit nucleotide substitution model for BI and ML analyses. For the *CCR* sequences, the best‐fit substitution model (GTR + I + G) was selected using the Akaike information criterion (AIC) in MrModeltest v2.3 (Nylander, 2004). The optimal partitioning scheme and the best‐fit nucleotide substitution model for each partition of the mtDNA genomes were estimated using the software PartitionFinder (Lanfear, Calcott, Ho, & Guindon, 2012). We defined the following sixteen partitions: 13 protein‐coding genes, two rRNAs, and one combined tRNA.

The BI analyses were performed using BEAST v 1.8.2 (Drummond & Rambaut, 2007). Three independent runs were performed for 100 million generations under a constant size and random starting topologies. The phylogenetic trees were sampled every 1,000th generation, which resulted in 100,000 trees, and the first 25% were discarded as burn‐ins. The effect sample sizes (ESSs) (>200) for the parameter estimates and convergence were checked with Tracer 1.5. (Rambaut & Drummond, 2007). The resulting trees were summarized in a Maximum Clade Credibility consensus tree with TreeAnnotator v1.8.2 (Drummond & Rambaut, 2007). The ML analyses were implemented in RAxML‐VI‐HPC (Stamatakis, 2006). Nodal support values were estimated from 1,000 nonparametric bootstrap replicates. The best partition scheme as determined by PartitionFinder was used for the mtDNA genome, respectively (Supporting Information Table S4 in Appendix S1).

To explore the haplotype genealogies for the nuDNA sequences, median‐joining networks were constructed in Network v4.6.1.0 (Bandelt, Forster, & Rohl, 1999) using the longest nonrecombining region.

### 2.4 Molecular diversity and population genetic structure

The molecular diversity of each population and clade with more than five individuals, including the number of haplotypes (*n*), haplotype diversity (*h*), and nucleotide diversity (*θ_π_* and *θ_ω_*), was estimated using DnaSP 5.10. Comparing estimates of current (*θ_π_*) and historical (*θ_ω_*) genetic diversity can provide genetic signatures into population dynamics over recent evolutionary history (Pearse & Crandall, 2004). Divergence between the clades was estimated using Kimura's (1980) two‐parameter (K2P) model implemented in MEGA 6.0. The two aforementioned analyses were calculated for *CCR* sequences. A Mantel test for detecting isolation‐by‐distance (IBD) pattern for *CCR* sequences was performed with Alleles In Space (AIS) (Miller, 2005).

To investigate the level of genetic variation among geographic populations, analyses of molecular variance (AMOVA; Excoffier, Smouse, & Quattro, 1992) were performed in Arlequin 3.5 (Excoffier & Lischer, 2010). Overall populations were sorted into geographic groups based on drainage (14 rivers) and mountain (nine mountains) systems (Supporting Information Table S1 in Appendix S1). Population differentiation (*ϕ*_ST_) was calculated in Arlequin by calculating pairwise *ϕ*_ST_ values among populations with more than five samples. We employed 1,000 permutations to assess significance for AMOVA and *ϕ*_ST_ calculations using *CCR* sequences based on K2P distance. The existence of phylogeographic structure was examined by calculating two genetic differentiation indices (*G*_ST_ and *N*_ST_) in DnaSP (Pons & Petit, 1996). *N*_ST_ > G_ST_ suggests strong relationship between phylogeny and geography.

### 2.5 Divergence time estimates

To obtain the dating for *A. davidianus* clades, we used a coalescent time estimation method in BEAST v 1.8.2 (Drummond & Rambaut, 2007) with mtDNA genome data only. As optimal partition strategy did not yield robust phylogenetic trees (data not shown), we selected no‐partition strategy for the mtDNA genome in the divergence time estimation. No‐partition strategy with the GTR + I + G model inferred by MrModeltest v2.3 and the uncorrelated lognormal relaxed clock (Drummond, Ho, Phillips, & Rambaut, 2006) were employed in this analysis. We used the isolation time (16 Ma) of Japan from mainland continental Eurasia (Isozaki, Aoki, Nakama, & Yanai, 2010), as the calibration point between Chinese and Japan giant salamanders. We set a lognormal prior to the tree root age, with 16.00 as the mean and 0.04 million years as standard deviation (95% CI: 14.97–17.07 Ma). The analysis was performed using 200 million generations and sampling every 2,000th tree under a Yule speciation prior and random starting topologies. The ESSs (>200) for the parameter estimates and convergence were checked with Tracer 1.5. Subsequently, after removing 25% of the resulting trees as burn‐in, the resulting trees were summarized in a Maximum Clade Credibility consensus tree with TreeAnnotator v1.8.2.

## 3 RESULTS

### 3.1 Phylogenetic analyses

**Figure 4:**
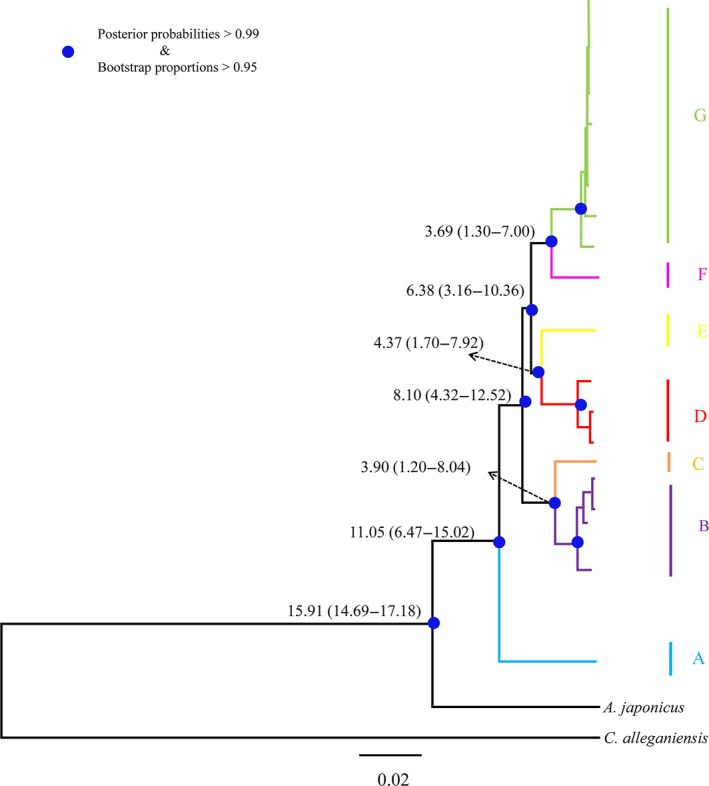
Bayesian tree based on mtDNA genomes for *Andrias davidianus*. Numbers near branches indicate divergence time estimates among the clades. Blue dots represent Bayesian posterior probabilities (>0.99) from Bayesian inferences and bootstrap proportions (>0.95) from maximum likelihood analyses in each clade. Colors correspond to those in Figure 3

The BI and ML analyses based on the *CCR* sequences consistently resolved five highly supported clades (A–G; Figure 3), but they did not yield well‐supported topologies in certain nodes. The BI tree performed strongly supported the clades F and G (Figure 3), whereas the ML trees did not distinguish these clades. The BI and ML analyses based on the concatenated dataset from the 19 mtDNA genomes obtained robust and similar topology (Figure 4). The ingroup species on the trees consistently contained seven major clades. Clade A was at the base of the tree and was the sister group of all other clades. Clades B and C, D, and E, and F and G were clustered together, respectively. Clade A included only members from locality 31 in Guangxi Province (Figure 2a). Clade B was primarily composed of specimens collected from Guizhou Province, northwestern Hunan Province, and Chongqing (Figure 2b). Clade C mainly occurred in the northern Guangxi Zhuang Autonomous Region and southern Hunan Province (Figure 2b). Clades D and E distributed in eastern parts of the species range and had a geographic occurrence in the two sides of Huangshan and Wuyi Mountains (Figure 2a). The clade F only detected in Tianshui County, Gansu Province, whereas the clade G occurred mainly in western and northern parts of the species range (Figure 2). The distribution pattern of major haplotype clades suggested that most populations tended to include a pure haplotypes (Figure 2). Only five populations, that is, localities 1, 13, 14, 16, and 18 were found to include haplotypes from two clades. In addition, we found that the Yangtze River basin held five clades (i.e., clades B, C, D, F, and G) and the Pearl River basin had three clades (i.e., clades A, B, and D) (Figure 2b; Supporting Information Figure S2b in Appendix S1). Both the Yellow River basin and southeastern Coastal River basin contained only one clade (Figure 2b; Supporting Information Figure S2c in Appendix S1).

Phylogeographic analyses showed that there are seven obviously geographic partitions of haplotype clades (Figures 2 and 3; Supporting Information Figure S2 in Appendix S1). Clades A, B, D, E, and F were distributed in closely geographic partitions, whereas clade G was distributed in two geographic partitions (northern and western species ranges). Some mountains, rivers, and provinces have more than one clades. Clades A and F were only detected in the Maoershan Mountain and the Qingling Mountain, respectively (Supporting Information Figure S2a in Appendix S1). Clades E distributed in eastern parts of the species range and had a geographic occurrence in the two sides of the Huangshan Mountain and the Wuyi Mountain (Supporting Information Figure S2a in Appendix S1). Clades B, C, D, and G were found in three, four, three, and seven mountains (Supporting Information Figure S2a in Appendix S1). The Yangzte River, the Yellow River, the Pearl River, and the Southern Coastal River habored five, one, three, and one clades, respectively (Figure 2b, Supporting Information Figure S2b in Appendix S1). More than one clades were examined in Hunan, Guangxi, Chongqing, and Gansu (Supporting Information Figure S2c in Appendix S1). Additionally, clade G had widest coverage that discovered in nine provinces.

The alignment of *RAG2* (772 bp) identified five haplotypes (A1–A5). The network of the *RAG2* sequences did not show any strong geographic patterning (Supporting Information Figure S3 in Appendix S1). The distribution patterns of the five alleles (Supporting Information Figure S3 in Appendix S1) did not show any obvious geographic patterning. The main allele A1 was present in almost all of the analyzed populations except in localities 4 and 12, which shared allele A2 (Supporting Information Figure S3 in Appendix S1). The A4 was shared by two populations (localities 17 and 26). The alleles A3 and A5 were private in locality 31 and locality 26, respectively.

### 3.2 Genetic diversity and population structure

The *CCR* results indicated high overall haplotype diversity (*h = *0.870) and nucleotide diversity (*θ_π_* = 0.230), respectively. However, there was considerable variation among different populations (Supporting Information Table S5 in Appendix S1). The population at locality 19 harbored the highest haplotype diversity with all haplotypes assigned to clade G. The nucleotide diversity was greatest in locality 16, and this population had haplotypes from clades C and G. Most populations displayed lower genetic diversity (Supporting Information Table S5 in Appendix S1); worse still, the values of *h *and *θ_π_* for six out of 23 populations equal to 0. Estimates of the current (*θ_π_*) and historical (*θ_ω_*) genetic diversity for each population indicated that 12 out of 23 populations showed a pattern of decline (*θ_π_* < *θ_ω_*; Supporting Information Table S1 in the Appendix S1). Same analysis conducted on different mtDNA clades showed that clades C, D, and G showed a pattern of decline (*θ_π_* < *θ_ω_*; Table 1). The genetic divergence among the seven clades based on the K2P distance model varied from 1.92% to 4.37% (Supporting Information Table S6 in Appendix S1). Mantel test detected weak but significant correlation between geographic and genetic distances (*r* = 0.242, *p* = 0.001).

**Table 1:** Genetic diversity for each clade

	*n*/*N*	*h*	*θ_π_* (%)	*θ_ω _*(%)
A	5/21	0.571 ± 0.052	0.200	0.123
B	9/67	0.525 ± 0.060	0.282	0.232
C	3/32	0.232 ± 0.094	**0.134**	**0.175**
D	8/24	0.692 ± 0.095	**0.239**	**0.432**
E	3/8	0.679 ± 0.122	0.078	0.073
F	1/1	—	—	—
G	41/167	0.638 ± 0.043	**0.292**	**0.828**
Overall	70/320	0.870 ± 0.015	2.2229	1.771

Notes

Bold values indicate *θ_π_* < *θ_ω_*.

*h*: haplotype diversity; *n*: haplotype numbers; *N*: individual numbers; *θ_ω_*: historical nucleotide diversity; *θ_π_*: current nucleotide diversity.

Nonhierarchical AMOVA (Table 2) suggested that *A. davidianus* was highly geographically structured, with 66.11% of the genetic variation attributable to differentiation among populations, a result which was highly significant (*p* < 0.001). Hierarchical AMOVA (Table 2) of populations partitioned according to river and mountain systems also demonstrated statistically significant differentiation (River systems: *ϕ*_CT_ = 0.597, *p* < 0.001; Mountain systems: *ϕ*_CT_ = 0.379, *p* < 0.001). A comparison of the fixation indices *N*_ST_ and *G*_ST_ revealed that *N*_ST_ was much larger than *G*_ST_ (0.726 and 0.510, respectively). The *ϕ*_ST_ calculations (data not shown) showed that 83.3% of the pairwise population comparisons were statistically significant (*p* < 0.05).

**Table 2:** Results of Analysis of Molecular Variance (AMOVA) for the two grouping options based on the mountain systems and drainages of the *Andrias davidianus* estimated using *ϕ*_ST_ based on *CCR *sequences

	Group compositions	Among groups (%)	Among populations within groups (%)	Within populations (%)	*F*_CT_	*p*
Overall populations	No group	66.11	—	33.89	0.661	<0.001
	Drainages	59.66	7.27	33.07	0.597	<0.001
	Mountains	37.94	29.48	32.59	0.379	<0.001

### 3.3 Divergence time estimates

The average estimates of the divergence time are shown in Figure 4. Clade A was estimated to have diverged at 11.05 Ma (95% CI 6.47–15.02 Ma). Major clades (clade B–C, D–E, and F–G) diverged between 6.38 Ma and 8.10 Ma (95% CI 3.16–10.36 to 4.32–12.52 Ma). The divergence between clades B and C occurred at approximately 3.90 Ma (95% CI 1.20–8.04 Ma), the divergence between clades D and E occurred at approximately 4.37 Ma (95% CI 1.70–7.92 Mya), and the divergence between clades F and G occurred at approximately 3.69 Ma (95% CI 1.30–7.00 Ma).

## 4 DISCUSSION

This study investigates the phylogeographic patterns of *A. davidianus *populations and presents some conservation implications for this endangered species based on a wide geographic sampling and using multiple markers. In addition, we compared our study and Yan et al. (2018)'s study in many aspects, for example, distribution and evolutionary relationships of observed clades, phylogeographic signals, and conservation implications. In comparison with Yan et al. (2018)'s study, our study revealed two new clades and more integrated distribution patterns of clades in natural range, and obtained more robust phylogenetic relationships among the clades. Moreover, a handful of phylogeographic signals and conservation implications regarding population and clade levels were firstly proposed.

### 4.1 Clades and their distribution patterns

Our findings demonstrate that presently wild‐caught *A. davidianus *is composed of seven highly divergent and strongly supported mtDNA clades in their natural species range. By contrast, Yan et al. (2018) recovered five clades from seventy wild‐caught samples using three mtDNA markers. However, Yan et al. (2018) determined seven clades from more than 1,000 farm‐bred individuals using three mtDNA markers, which was in line with our findings. Given that relatively small sample size and limited sample coverage of wild‐caught individuals used in Yan et al. (2018), our study may comprehensively outline the genetically current situation of *A. davidianus *populations in natural species ranges.

To compare the two studies conveniently, we conducted one‐to‐one correspondence for the clades (our study–study by Yan et al. (2018): A–A, B–D, C–U1, D–U2, E–E, F–C, and G–B) (Figure 3). Clades C and D, missed in Yan et al. (2018) using seventy wild‐caught samples and only observed in farm, were firstly reported in natural species range in our study. The members of clade C located in northern Guangxi and center Hunan, and the representatives of clade D occurred in western Jiangxi (localities 23 and 32) and northern Guangdong Province (locality 33). The basal clade A only occurred in northern Guangxi was highly consistent with the study by Yan et al. (2018). With regard to clade B, apart from living in Guizhou and Chongqing (Yan et al., 2018), we found that this clade also located in northwestern Hunan (Figure 2). Clade D, discovering in southern Anhui (locality 24) in Yan et al. (2018), was also discovered in central Zhejiang (locality 25) in our study. Clade F was reported occurred in northeastern Sichuan in Yan et al. (2018), while it was uncovered in southern Gansu in our study. This case was not unexpected as these two locations are both belonged to the Jialingjiang River and geographic proximity. Additionally, our study observed clade G mainly occupied western, center, and northern species range, including Yunnan, Sichuan, Gansu, Shaanxi, Shanxi, Henan, Hubei, and northern Hunan, which was partly concordant with the study by Yan et al. (2018) and greatly enlarged the distribution ranges of clade G. The distributions of this clade detected in central species range (i.e., Hubei and northern Hunan) were firstly reported. Our results, to this end, showed a relatively intact and thorough distribution pattern of distinct *A. davidianus* clades in natural species range.

### 4.2 Discordant phylogenetic relationships among clades

The phylogetic analyses of mtDNA genomes in our study supported four major clades within *A. davidianus* populations: (a) clade A; (b) clade B–C; (c) clade D–E; and (d) clade F–G. Our analyses showed clade D–E and clade F–G clustered together and clade B–C was supported to be sister to the two aforementioned major clades. This result contradicted the outcome demonstrated in Yan et al. (2018). Yan et al. (2018) suggested that clade B–C and clade D–E were firstly clustered into a clade and clade F–G was sister to the mixed clade with low supported values. Different sequence lengths used in the two studies might cause this controversial outcome. Adequate sequence lengths are of importance to obtain strong phylogenetic inference among different taxa, particularly among closely related species groups (McHardy, Martin, Tsirigos, Hugenholtz, & Rigoutsos, 2007). For instance, we failed to acquire robust phylogenetic relationships among the clades B‐G using *CCR* sequences (<1,600 bp), though seven clades were highly resolved. Three mtDNA genes employed in Yan et al. (2018) seemed not enough as they did not gain high supported value in the node containing clade B–C, clade D–E, and clade F–G. Thus, exploring more molecular markers is critically necessary to further resolve the controversial phylogenetic relationship among *A. davidianus* clades.

### 4.3 Phylogeographic patterns

A relatively high level of mtDNA genetic differentiation (1.92% to 4.37%) was observed among the seven clades. The high levels of genetic diversity uncovered in *A. davidianus* are not unexpected since previous genetic studies of the congeneric *A. japonicus* and confamilial *C. alleganiensis* also revealed detectable intraspecific divergence (Matsui, Tominaga, Liu, & Tanaka‐Ueno, 2008; Sabatino & Routman, 2009). For instance, Matsui et al. (2008) detected that *A. japonicus* populations were divided into two clades with 1.1% sequence divergence between them. Likewise, Sabatino and Routman (2009) found that *C. alleganiensis *populations in eight reciprocally monophyletic clades had 0.7% to 5.4% sequence divergence between them.

The divergence observed within *A. davidianus* likely reflects long‐term isolation. The divergence between the major clades (A, B–C, D–E, and F–G) was estimated to have occurred 6.38 to 11.05 Mya in the late Miocene during the intense uplift phase of the Qinghai‐Tibetan Plateau (An et al., 2001; Cui et al., 1996). The split times between clades B and C, between D and E, and between F and G ranged from 3.69 to 4.37 Mya, indicating that these clades separated during the middle Pliocene and fitted with the rapid and drastic uplifting of the Qinghai‐Tibetan Plateau (An et al., 2001; Cui et al., 1996; Li et al., 2001; Zheng et al., 2000). The divergence time estimates were largely agreed with the results calculated in Yan et al. (2018). During these periods, many mountains rose and drainage systems were rearranged in East Asia (He & Chen, 2006; Li & Fang, 1999; Shi, Li, & Li, 1998). Considering that *A. davidianus* is a fully aquatic species and dispersal events across mountains are unlikely, separated drainage systems and uplifted mountains attributed to tectonic events almost certainly caused the differentiation of *A. davidianus *clades. High and statistically significant differentiation of *A. davidianus* populations according to river systems and mountain systems inferred from AMOVA provides evidence to support this hypothesis. For example, although locality 30 (clade C) and locality 31 (clade A) occur in close spatial proximity in Maoershan Mountains, these populations belong different mtDNA clades because they reside on different drainage systems opposite sides of the mountain system (Supporting Information Table S1 and Figure S4a in Appendix S1). Similar case was observed in localities 15, 16, 18, and 19. Localities 15 and 18 (clade B) and localities 16 and 19 (clade G) occur in close spatial proximity in Wuling Mountains, but these populations belong different mtDNA clades because they live in different rivers (localities 15 and 18 belong to Lishui River and localities 16 and 19 belong to Yuanjiang River) (Supporting Information Table S1 and Figure S4b in Appendix S1). Furthermore, the Huangshan and Wuyi Mountains in southeastern China may act as phylogeographic barriers to block gene exchange between clades D and E (Figure 2a). Likewise, the Xuefengshan Mountains in Hunan seem to separate clades B and C (Figure 2a). Number of earlier studies had argued that the drastic uplift of the plateau largely re‐shaped the landscape features of central and eastern Asia, and have been hypothesized as an important driving force of vicariant speciation and intraspecific divergence in many amphibian species (Che et al., 2010; Lu, Zheng, Murphy, & Zeng, 2012; Macey et al., 1998; Yan et al., 2013; Zhou et al., 2012). Thus, the complex geological history appears to be an important factor for driving the divergence of *A. davidianus* clades.

In addition to the influence of tectonic activity, specific habitat requirements, natural barriers (i.e., mountains and rivers), and poor dispersal ability likely have influenced patterns and level of divergence among *A. davidianus* populations. Although the main native distribution area of this species is in pristine mountain rivers and streams at elevations ranging from 200 to 2,000 m (Fu, 1993), the majority of the sampled populations were distributed in rocky montane streams at elevations ranging from 300 to 900 m. Yet, the AMOVA and *ϕ*_ST_ analyses indicated a high level of genetic structuring among *A. davidianus* populations. *N*_ST_ > *G*_ST_ indicated that a strong relationship between phylogeny and geography had been inferred within *A. davidianus*. Further evidence for geographically ordered genetic structuring was provided by the restricted geographic distribution of certain clades (Figure 2a). For example, clades B and C occur mainly in the southern and center part of the species range, while clades D and E occur almost exclusively in the southeastern part of the species range. Moreover, *A. davidianus* clades are surrounded by a large number of mountains and belonged to different rivers (Figure 2). Mountains and rivers may form natural barriers to block migration. The significant correlation between geographic and genetic distances suggested that poor dispersal potential may be a considerable factor that triggered the fairly high degree of population differentiation.

The analyses of *RAG2* sequences uncovered high level of allele sharing among the seven clades. Compared to relatively obvious geographic structuring of the mtDNA clades, the distribution of the five *RAG2* alleles displayed no obvious geographic structuring. Widespread and random distribution of alleles A1 and A2 in each population suggests that incomplete lineage sorting may explain the discrepancy between the topologies generated from mtDNA and nuDNA sequences.

### 4.4 Insights for *A. davidianus *conservation

The Chinese giant salamander is the largest living amphibian species in the world. Contemporary populations of *A. davidianus* face many threats from anthropogenic activities, such as habitat destruction, water pollution, and poaching (Dai et al., 2009; Zhang, Wang, Wu, Wang, & Huang, 2002). The results of this study provide some insights that might help improving monitoring programs and developing conservation strategies for this species.

The maintenance of genetic diversity is of critical importance for conserving the evolutionary potential of a given species (Milligan, Leebensmack, & Strand, 1994). Yan et al. (2018) did not estimate the genetic diversity of population and clade level due to limitation in sample size. Although our study detected high levels of genetic diversity in whole wild‐caught *A. davidianus* populations, signals of genetic diversity decline were also discovered when comparing estimates of current and historical genetic diversity for each population and clade. Twelve populations and three clades (C, D, and G) showed a pattern of genetic diversity decline. Thus, there are indications that at least some *A. davidianus* populations are losing genetic variation, and these trends should be followed up. Likewise, the population in locality 26 might be worth protection because of its genetic uniqueness of carrying allele A4. The highest priority for conservation should be allocated to the population in locality 31, which is the only known representative of the clade A and allele A3, which is also the indicated to be oldest of the seven detected mtDNA clades.

Human‐assisted translocations have had an unknown influence on genetic diversity and structuring of wild *A. davidianus* populations. Although the release of captive‐reared individuals to wild could have a positive effect on *A. davidanus* populations, also negative effects are possible. For instance, the introduction of individuals with non‐native genotypes might lead to loss of local adaptations and native genetic variability. Similarly, supplementation of wild populations with captive breed individuals might lead to Ryman–Laikre effect (Ryman & Laikre, 1991), which refers to reduction in the effective size of the supplemented population due to increased variance in family size. Hence, since both the ecological and genetic consequences of introducing *A. davidanus* individuals of captive origin on native populations are largely unknown, research into the potential effects of this supportive breeding on local populations is needed. Seven divergent mitochondrial clades were found, and some mountains, rivers, and provinces had more than one clades, so we suggest that genetic lineage testing is needed before artificial release, and released individuals should have the same lineage to native ones. At any rate, the results of this study provide a backbone for future research efforts directed toward generating a management and conservation plans with a strong scientific and ethical basis for this species of special interest.

## CONFLICT OF INTEREST

None declared.

## AUTHOR CONTRIBUTIONS

Z.Q.L., D.Q.W., Q.W.W., W.T.C, and S.P.H. designed the research. Z.Q.L., C.R.W., Y.A.W., P.H., J.X., and C.H.L performed the sampling and analyzed field data. Z.Q.L., D.Q.W., and S.H.Z performed the laboratory analyses. W.T.C., D.Q.W., and Z.Q.L. wrote the initial manuscript. J.M edited the language of the initial manuscript and provided many suggestions. Funding support was provided by Q.W.W. (Grant Nos. No. 201203086) and D.Q.W (Grant Nos. LFBC0809).

## DATA ACCESSIBILITY

DNA sequences have been deposited in GenBank under Accession numbers KU131040, KU131040–KU131048, KU131050–KU131059, KU131061–KU131066, KU131070–KU131071, KU131074–KU131075, KU131080–KU131082, KU131084, KU131090, KU131093, KU131096–KU131101, KU131105–KU131107, KU131110–KU131111, KU131113, KU131117–KU131122, KU131125, KU131131–KU131143, KU131145–KU131153, KU131163–KU131166, KU131168–KU131170, KU131172, KU131174–KU131179, KU131184–KU131187, KX503809–KX503810, and KX503816. Details regarding individual samples are available in Supporting Information Table S1 in Appendix S1.
