# Supplementary material for: Phylogeographic patterns and conservation implications of the endangered Chinese giant salamander
Source: Ecol Evol. 2019 Mar 7;9(7):3879–90. doi: 10.1002/ece3.5014 (PMC6467858; doi:10.1002/ece3.5014)
Supplement: Supplementary file 1 [file ECE3-9-3879-s001.docx]

**Phylogeographic patterns and conservation implications of the endangered Chinese giant salamander**

Zhi-Qiang Liang^1,2,3,4*^, Wei-Tao Chen^5,6*^, Shu-Huan Zhang^1^, Chong-Rui Wang^2^, Shun-Ping He^5^, Yuan-An Wu^2,3^, Ping He^7^, Jiang Xie^1^, Chuan-Wu Li^2,3^, Juha Merilä^8^, Deng-Qiang Wang^1**^ and Qi-Wei Wei^1,4**^

^1^Key Laboratory of Freshwater Biodiversity Conservation, Ministry of Agriculture of China, Yangtze River Fisheries Research Institute, Chinese Academy of Fishery Sciences, Wuhan, Hubei, China

^2^Hunan Fisheries Science Institute, Changsha, Hunan, China

^3^Collaborative Innovation Center for Efficient and Health Production of Fisheries in Hunan Province, Changde, Hunan, China

^4^College of Life Science, Southwest University, Chongqing, China

^5^The Key Laboratory of Aquatic Biodiversity and Conservation of Chinese Academy of Sciences, Institute of Hydrobiology, Chinese Academy of Sciences, Wuhan, China

^6^Pearl River Fisheries Research Institute, Chinese Academy of Fishery Science, Guangzhou, China

^7^Administrative Office of The National Giant Salamander Nature Reserve of Zhangjiajie, Hunan, China

^8^Ecological Genetics Research Unit, Research Programme in Organismal and Evolutionary Biology, Faculty of Biological and Environmental Sciences, Department of Biosciences, University of Helsinki, Helsinki, Finland

^*^authors contributed equally to this work.

^**^Corresponding author: Dengqiang Wang, Fax: +8602781780118, E-mail: wdq@yfi.ac.cn and Qiwei Wei, Fax: +862781780118, E-mail: weiqw@yfi.ac.cn. Address: Key Laboratory of Freshwater Biodiversity Conservation, Ministry of Agriculture of China, Yangtze River Fisheries Research Institute, Chinese Academy of Fishery Sciences, Wuhan, 430223, Hubei, China

**Supporting information**

**Table S1:** Detailed information for specimens of for *Andrias davidianus* in this study. The distributions of the *A*. *davidianus* are shown in Fig. 1. Locality numbers correspond to Fig. 1. Locality, coordinates (latitude/longitude), sample size, voucher number, *CCR* haplotype and GenBank accession number for *CCR*, mtDNA genome and *RAG-2* are presented.Tissue abbreviations are: F = caudal fin clips; O = oral mucosa cells; S = exfoliated skin cuticle.

**Table S2**: Sample information from ten natural breeding caves of *A. davidianus*. Abbreviations are: a, natural breeding caves of *A. davidianus* were reported; b, natural breeding caves that were not reported before this paper; c, sampled adults grew up from the cave larvae.

**Table S2**: Information of primer pairs used in our study.

**Table S3**: Optimal partitioning scheme and best-fit nucleotide substitution model for each partition of the mtDNA genomes as estimated with the software PartitionFinder.

**Table S4**: Genetic diversity for each population using *CCR* sequences. Localities are detailed in Table S1. N, individual numbers; n, haplotype numbers; *h*, haplotype diversity; ɵ_π_, current nucleotide diversity; ɵ*_w_*, historical nucleotide diversity. Bold values indicate ɵ_π <_ ɵ*_w_*.

**Table S5:** Genetic distance based on *CCR* sequences among the seven clades estimated by K2P distance.

**Figure S1:** Wild-caught larvae and adults of *A. davidianus*. a, b, c, larvae from Yuanzi Cave in Zhangjiajie. Time of the larva left from the cave for the first time: 1, 15 h; 2, 5 h; 3, 20 min; 4, 50 min; 5, 60 min; 6, 30 min; 7, 12 min. d, e, adults from Jinggangshan and Zhangjiajie, respectively, and their skin speckle characteristics are netlike and block, respectively. Larva 3, 6, 7 is the same one. Sample number of larva 1-5, 8,9 is HNSZSDJ78-82, JXJGS352, HNSM291, respectively. Inset place in upper right corner each picture shows the source of the sample individuals.

**Figure S2:** Map showing the sampling clades of *Andrias davidianus* based on mountains (a), rivers (b) and provinces (c).

**Figure S3**: Map showing the distribution of *RAG2* alleles in *Andrias davidianus* populations. Localities of pie-diagrams correspond to Fig. 3. The upper left map is median-joining network of nuclear gene alleles for *RAG2* gene and colors correspond to those in Fig. 3.

**Figure S4:** Map showing the sampling clades of *Andrias davidianus* based on mountains and rivers in Ziyuan, Xingan, Lianzhou (a) and Zhangjiajie (b). Inset in lower left corner shows the simplified maternal genealogy with clades A–G. Colors of pie-diagrams and tree correspond to the clades in Fig. 3.

**Table S1**

| **Locality (number)** | **Mountain (M.)** | **River (R.)** | **Longitude** | **Latitude** | **Tissue** | **Sample** | **Voucher No.** | **Clade** | **GenBank nos** | | |
| --- | --- | --- | --- | --- | --- | --- | --- | --- | --- | --- | --- |
|  |  |  |  |  |  | **size** |  |  | ***CCR*** | ***Mt* genome** | ***RAG-2*** |
| **Qinzhou, Tianshui, Gansu (** **1)** | Qinling, Qingba M. | Baijiahe, Jialingjiang, Yangzte R. | 105.9E | 34.2N | F, S | 6 | GSTS239 | G | KU131179 |  |  |
|  |  |  |  |  |  |  | GSTS240 | F | KU131184 | KU131042 | KU131185 |
|  |  |  |  |  |  |  | GSTS270 | G | KU131139 |  |  |
|  |  |  |  |  |  |  | GSTS1015 | G | KU131118 |  |  |
|  |  |  |  |  |  |  | GSTS1016 | G | KU131118 |  |  |
|  |  |  |  |  |  |  | GSTS1017 | G | KU131118 |  |  |
| **Yinge, Taibai, Baoji, Shaanxi (2)** | Qinling, Qingba M. | Shitouhe, Weihe, Yellow R. | 107.6E | 34.1N | F, O | 2 | SXTBYG677 | G | KU131118 | KU131058 |  |
|  |  |  |  |  |  |  | SXTBYG678 | G | KU131125 |  |  |
| **Taibaihe, Taibai, Baoji, Shaanxi (3)** | Qinling, Qingba M. | Taibaihe, Hanjiang, Yangzte R. | 107.5E | 33.7N | F, O | 23 | SXTB201 | G | KU131168 |  | KU131185 |
|  |  |  |  |  |  |  | SXTB202 | G | KU131168 | KU131040 |  |
|  |  |  |  |  |  |  | SXTB203 | G | KU131168 |  |  |
|  |  |  |  |  |  |  | SXTB204 | G | KU131119 |  | KU131185 |
|  |  |  |  |  |  |  | SXTB205 | G | KU131169 |  | KU131185 |
|  |  |  |  |  |  |  | SXTB206 | G | KU131119 |  |  |
|  |  |  |  |  |  |  | SXTB207 | G | KU131119 |  |  |
|  |  |  |  |  |  |  | SXTB208 | G | KU131118 |  |  |
|  |  |  |  |  |  |  | SXTB209 | G | KU131118 |  | KU131185 |
|  |  |  |  |  |  |  | SXTB210 | G | KU131118 |  |  |
|  |  |  |  |  |  |  | SXTB211 | G | KU131119 |  |  |
|  |  |  |  |  |  |  | SXTB212 | G | KU131118 |  |  |
|  |  |  |  |  |  |  | SXTB213 | G | KU131118 |  |  |
|  |  |  |  |  |  |  | SXTB215 | G | KU131118 |  |  |
|  |  |  |  |  |  |  | SXTB216 | G | KU131118 |  |  |
|  |  |  |  |  |  |  | SXTB217 | G | KU131118 |  |  |
|  |  |  |  |  |  |  | SXTB218 | G | KU131122 |  |  |
|  |  |  |  |  |  |  | SXTB219 | G | KU131118 |  |  |
|  |  |  |  |  |  |  | SXTB220 | G | KU131119 |  |  |
|  |  |  |  |  |  |  | SXTB221 | G | KU131119 |  | KU131185 |
|  |  |  |  |  |  |  | SXTB222 | G | KU131118 |  |  |
|  |  |  |  |  |  |  | SXTB223 | G | KU131118 |  |  |
|  |  |  |  |  |  |  | SXTB224 | G | KU131118 |  |  |
| **Zhashui, Shangluo, Shaanxi (4)** | Qinling, Qingba M. | Qianyouhe, Hanjiang, Yangzte R. | 109.2E | 33.8N | O | 9 | SXSLZS666 | G | KU131170 |  |  |
|  |  |  |  |  |  |  | SXSLZS667 | G | KU131170 |  | KU131186 |
|  |  |  |  |  |  |  | SXSLZS668 | G | KU131170 |  |  |
|  |  |  |  |  |  |  | SXSLZS670 | G | KU131170 |  | KU131186 |
|  |  |  |  |  |  |  | SXSLZS671 | G | KU131170 |  | KU131186 |
|  |  |  |  |  |  |  | SXSLZS672 | G | KU131170 | KU131057 |  |
|  |  |  |  |  |  |  | SXSLZS673 | G | KU131170 |  |  |
|  |  |  |  |  |  |  | SXSLZS674 | G | KU131170 |  |  |
|  |  |  |  |  |  |  | SXSLZS676 | G | KU131170 |  | KU131186 |
| **Xihonghong, Lishan, Yuanqu, Shanxi (5)** | Lishan, Zhongtiaoshan M. | Xiyanghe, Yellow R. | 112.1E | 35.3N | O | 14 | SXYQLS368 | G | KU131145 |  | KU131185 |
|  |  |  |  |  |  |  | SXYQLS369 | G | KU131119 |  |  |
|  |  |  |  |  |  |  | SXYQLS370 | G | KU131146 |  |  |
|  |  |  |  |  |  |  | SXYQLS371 | G | KU131118 | KU131047 |  |
|  |  |  |  |  |  |  | SXYQLS372 | G | KU131118 |  |  |
|  |  |  |  |  |  |  | SXYQLS373 | G | KU131118 |  |  |
|  |  |  |  |  |  |  | SXYQLS374 | G | KU131118 |  |  |
|  |  |  |  |  |  |  | SXYQLS376 | G | KU131118 |  |  |
|  |  |  |  |  |  |  | SXYQLS377 | G | KU131118 |  | KU131185 |
|  |  |  |  |  |  |  | SXYQLS378 | G | KU131147 |  | KU131185 |
|  |  |  |  |  |  |  | SXYQLS380 | G | KU131148 |  | KU131185 |
|  |  |  |  |  |  |  | SXYQLS381 | G | KU131118 |  | KU131186 |
|  |  |  |  |  |  |  | SXYQLS383 | G | KU131149 |  |  |
|  |  |  |  |  |  |  | SXYQLS384 | G | KU131118 |  | KU131185 |
| **Wangwoshan, Jiyuan, Henan (6)** | Wangwoshan, Zhongtiaoshan M. | Dayuhe, Yellow R. | 112.3E | 35.2N | O, S | 17 | HNJY385 | G | KU131119 |  | KU131186 |
|  |  |  |  |  |  |  | HNJY386 | G | KU131119 |  | KU131185 |
|  |  |  |  |  |  |  | HNJY387 | G | KU131119 |  | KU131185 |
|  |  |  |  |  |  |  | HNJY388 | G | KU131119 |  | KU131185 |
|  |  |  |  |  |  |  | HNJY389 | G | KU131119 |  |  |
|  |  |  |  |  |  |  | HNJY390 | G | KU131118 | KU131048 |  |
|  |  |  |  |  |  |  | HNJY392 | G | KU131119 |  |  |
|  |  |  |  |  |  |  | HNJY391 | G | KU131118 |  |  |
|  |  |  |  |  |  |  | HNJY394 | G | KU131118 |  | KU131186 |
|  |  |  |  |  |  |  | HNJY397 | G | KU131118 |  | KU131185 |
|  |  |  |  |  |  |  | HNJY398 | G | KU131118 |  |  |
|  |  |  |  |  |  |  | HNJY399 | G | KU131119 |  |  |
|  |  |  |  |  |  |  | HNJY403 | G | KU131118 |  |  |
|  |  |  |  |  |  |  | HNJY405 | G | KU131118 |  |  |
|  |  |  |  |  |  |  | HNJY406 | G | KU131118 |  |  |
|  |  |  |  |  |  |  | HNJY407 | G | KU131118 |  |  |
|  |  |  |  |  |  |  | HNJY408 | G | KU131118 |  |  |
| **Zhushan, Shiyan, Hubei ( 7 )** | Dabashan, Qingba M. | Duhe, Hanjiang, Yangzte R. | 109.6E | 32.3N | O | 7 | HBZS1031 | G | KU131118 |  |  |
|  |  |  |  |  |  |  | HBZS1034 | G | KU131118 |  |  |
|  |  |  |  |  |  |  | HBZS1035 | G | KU131118 |  |  |
|  |  |  |  |  |  |  | HBZS1036 | G | KU131118 |  |  |
|  |  |  |  |  |  |  | HBZS1037 | G | KU131118 |  |  |
|  |  |  |  |  |  |  | HBZS1038 | G | KU131118 |  |  |
|  |  |  |  |  |  |  | HBZS1039 | G | KU131118 |  |  |
| **Songbai, Shenlongjia, Hubei (8)** | Dabashan, Qingba M. | Xiangxihe, Yangzte R. | 110.7E | 31.7N | O | 1 | HBSLJ511 | G | KU131111 |  |  |
| **Mabian, Leshan, Sichuan (9)** | Daliangshan M. | Mabianhe, Minjiang , Yangzte R. | 103.5E | 28.8N | S | 6 | SCMB242 | G | KU131140 |  |  |
|  |  |  |  |  |  |  | SCMB243 | G | KU131119 |  | KU131185 |
|  |  |  |  |  |  |  | SCMB244 | G | KU131141 | KU131043 |  |
|  |  |  |  |  |  |  | SCMB245 | G | KU131141 |  |  |
|  |  |  |  |  |  |  | SCMB246 | G | KU131140 |  |  |
|  |  |  |  |  |  |  | SCMB247 | G | KU131141 |  | KU131186 |
| **Niujie, Yiliang, Zhaotong, Yunnan (10)** | Wumengshan M. | Baishuijiang, Hengjiang, Yangzte R. | 104.5E | 27.8N | O | 11 | YNYL550 | G | KU131150 |  |  |
|  |  |  |  |  |  |  | YNYL551 | G | KU131151 | KU131053 |  |
|  |  |  |  |  |  |  | YNYL552 | G | KU131152 |  | KU131185 |
|  |  |  |  |  |  |  | YNYL553 | G | KU131152 |  | KU131185 |
|  |  |  |  |  |  |  | YNYL554 | G | KU131119 |  | KU131185 |
|  |  |  |  |  |  |  | YNYL555 | G | KU131118 |  | KU131186 |
|  |  |  |  |  |  |  | YNYL556 | G | KU131140 |  |  |
|  |  |  |  |  |  |  | YNYL557 | G | KU131140 |  |  |
|  |  |  |  |  |  |  | YNYL558 | G | KU131140 |  | KU131186 |
|  |  |  |  |  |  |  | YNYL559 | G | KU131140 |  |  |
|  |  |  |  |  |  |  | YNYL561 | G | KU131118 |  |  |
| **Zhenxiong, Zhaotong, Yunan (11)** | Wumengshan M. | Hengjiang, Yangzte R | 104.5E | 27.5N | O | 10 | YNZX562 | G | KU131118 |  |  |
|  |  |  |  |  |  |  | YNZX563 | G | KU131118 |  |  |
|  |  |  |  |  |  |  | YNZX564 | G | KU131153 |  | KU131185 |
|  |  |  |  |  |  |  | YNZX565 | G | KU131118 |  | KU131185 |
|  |  |  |  |  |  |  | YNZX566 | G | KU131118 |  | KU131185 |
|  |  |  |  |  |  |  | YNZX567 | G | KU131118 |  |  |
|  |  |  |  |  |  |  | YNZX568 | G | KU131118 |  | KU131186 |
|  |  |  |  |  |  |  | YNZX569 | G | KU131118 |  | KU131185 |
|  |  |  |  |  |  |  | YNZX570 | G | KU131118 |  |  |
|  |  |  |  |  |  |  | YNZX571 | G | KU131118 |  | KU131186 |
| **Giant Salamander Hole, Daba, Xingwen, Yibin, Sichuan (12)** | Wumengshan M. | Yongninghe, Yangzte R | 105.1E | 28.1N | O | 17 | SCYB249 | G | KU131142 |  |  |
|  |  |  |  |  |  |  | SCYB251 | G | KU131143 |  |  |
|  |  |  |  |  |  |  | SCYB252 | G | KU131118 |  |  |
|  |  |  |  |  |  |  | SCYB253 | G | KU131119 |  |  |
|  |  |  |  |  |  |  | SCYB254 | G | KU131143 |  |  |
|  |  |  |  |  |  |  | SCYB255 | G | KU131143 |  | KU131186 |
|  |  |  |  |  |  |  | SCYB256 | G | KU131143 |  |  |
|  |  |  |  |  |  |  | SCYB257 | G | KU131143 |  |  |
|  |  |  |  |  |  |  | SCYB258 | G | KU131143 |  |  |
|  |  |  |  |  |  |  | SCYB259 | G | KU131143 |  |  |
|  |  |  |  |  |  |  | SCYB260 | G | KU131143 |  |  |
|  |  |  |  |  |  |  | SCYB261 | G | KU131143 |  |  |
|  |  |  |  |  |  |  | SCYB262 | G | KU131143 |  |  |
|  |  |  |  |  |  |  | SCYB263 | G | KU131143 |  |  |
|  |  |  |  |  |  |  | SCYB264 | G | KU131143 |  |  |
|  |  |  |  |  |  |  | SCYB265 | G | KU131143 |  |  |
|  |  |  |  |  |  |  | SCYB266 | G | KU131143 |  |  |
| **Longtan, Fuling, Chongqing (13)** | Daloushan M. | Longtanhe,Youjianghe, Yangzte R. | 107.1E | 29.4N | O | 13 | CQFL488 | G | KU131138 |  |  |
|  |  |  |  |  |  |  | CQFL489 | B | KU131074 |  |  |
|  |  |  |  |  |  |  | CQFL490 | B | KU131075 |  |  |
|  |  |  |  |  |  |  | CQFL491 | B | KU131075 |  |  |
|  |  |  |  |  |  |  | CQFL492 | B | KU131075 |  | KU131185 |
|  |  |  |  |  |  |  | CQFL493 | B | KU131075 |  |  |
|  |  |  |  |  |  |  | CQFL494 | B | KU131075 |  |  |
|  |  |  |  |  |  |  | CQFL495 | B | KU131075 |  | KU131185 |
|  |  |  |  |  |  |  | CQFL496 | B | KU131075 |  |  |
|  |  |  |  |  |  |  | CQFL497 | B | KU131075 |  |  |
|  |  |  |  |  |  |  | CQFL498 | B | KU131075 |  |  |
|  |  |  |  |  |  |  | CQFL499 | B | KU131075 |  |  |
|  |  |  |  |  |  |  | CQFL500 | B | KU131075 |  |  |
| **Huolu, Wulong, Chongqing (14)** | Daloushan M. | Wujiang, Yangzte R. | 107.9E | 29.3N | O | 8 | CQWL480 | B | KU131074 |  |  |
|  |  |  |  |  |  |  | CQWL481 | B | KU131064 | KU131051 |  |
|  |  |  |  |  |  |  | CQWL482 | B | KU131074 |  |  |
|  |  |  |  |  |  |  | CQWL483 | B | KU131064 |  |  |
|  |  |  |  |  |  |  | CQWL484 | B | KU131074 |  | KU131185 |
|  |  |  |  |  |  |  | CQWL485 | B | KU131074 |  | KU131185 |
|  |  |  |  |  |  |  | CQWL486 | B | KU131064 |  |  |
|  |  |  |  |  |  |  | CQWL487 | G | KU131110 |  | KU131185 |
| **Shuitianba Cave, Longshan, Xiangxi, Hunan (15)** | Wulingshan M. | Mengdonghe, Yuanjiang, Yangzte R. | 109.7E | 29.3N | O | 20 | HNLS54 | B | KU131064 |  |  |
|  |  |  |  |  |  |  | HNLS55 | B | KU131064 | KU131052 |  |
|  |  |  |  |  |  |  | HNLS56 | B | KU131064 |  |  |
|  |  |  |  |  |  |  | HNLS57 | B | KU131064 |  |  |
|  |  |  |  |  |  |  | HNLS58 | B | KU131064 |  |  |
|  |  |  |  |  |  |  | HNLS59 | B | KU131064 |  |  |
|  |  |  |  |  |  |  | HNLS61 | B | KU131064 |  |  |
|  |  |  |  |  |  |  | HNLS62 | B | KU131064 |  |  |
|  |  |  |  |  |  |  | HNLS63 | B | KU131064 |  |  |
|  |  |  |  |  |  |  | HNLS66 | B | KU131064 |  |  |
|  |  |  |  |  |  |  | HNLS67 | B | KU131062 |  |  |
|  |  |  |  |  |  |  | HNLS68 | B | KU131064 |  |  |
|  |  |  |  |  |  |  | HNLS69 | B | KU131064 |  |  |
|  |  |  |  |  |  |  | HNLS70 | B | KU131064 |  |  |
|  |  |  |  |  |  |  | HNLS71 | B | KU131062 |  |  |
|  |  |  |  |  |  |  | HNLS72 | B | KU131064 |  |  |
|  |  |  |  |  |  |  | HNLS73 | B | KU131064 |  |  |
|  |  |  |  |  |  |  | HNLS74 | B | KU131064 |  |  |
|  |  |  |  |  |  |  | HNLS75 | B | KU131064 |  |  |
|  |  |  |  |  |  |  | HNLS76 | B | KU131064 |  |  |
| **Hekou Cave, Sangzhi, Zhangjiajie, Hunan (16)** | Wulingshan M. | Lishui, Yangzte R. | 109.9E | 29.6N | O | 10 | HNSZHK312 | G | KU131118 |  | KU131185 |
|  |  |  |  |  |  |  | HNSZHK313 | G | KU131118 |  |  |
|  |  |  |  |  |  |  | HNSZHK314 | G | KU131120 |  |  |
|  |  |  |  |  |  |  | HNSZHK315 | C | KU131084 |  |  |
|  |  |  |  |  |  |  | HNSZHK316 | G | KU131165 |  | KU131185 |
|  |  |  |  |  |  |  | HNSZHK317 | C | KU131084 |  | KU131185 |
|  |  |  |  |  |  |  | HNSZHK318 | C | KU131084 |  | KU131186 |
|  |  |  |  |  |  |  | HNSZHK319 | G | KU131118 |  | KU131186 |
|  |  |  |  |  |  |  | HNSZHK320 | G | KU131166 |  | KU131185 |
|  |  |  |  |  |  |  | HNSZHK116 | G | KU131164 |  |  |
| **Hupingshan Cave, Shimen, Changde, Hunan (17)** | Wulingshan M. | Beixihe, Lishui,Yangzte R. | 110.7E | 30.0N | O | 21 | HNSM291 | G | KU131118 |  | KX503809 |
|  |  |  |  |  |  |  | HNSM292 | G | KU131119 |  |  |
|  |  |  |  |  |  |  | HNSM293 | G | KU131118 |  |  |
|  |  |  |  |  |  |  | HNSM294 | G | KU131118 |  |  |
|  |  |  |  |  |  |  | HNSM295 | G | KU131118 |  |  |
|  |  |  |  |  |  |  | HNSM296 | G | KU131163 |  | KU131185 |
|  |  |  |  |  |  |  | HNSM297 | G | KU131118 |  | KX503809 |
|  |  |  |  |  |  |  | HNSM298 | G | KU131118 |  | KU131185 |
|  |  |  |  |  |  |  | HNSM299 | G | KU131118 |  | KU131185 |
|  |  |  |  |  |  |  | HNSM300 | G | KU131118 |  |  |
|  |  |  |  |  |  |  | HNSM301 | G | KU131118 |  |  |
|  |  |  |  |  |  |  | HNSM302 | G | KU131118 |  |  |
|  |  |  |  |  |  |  | HNSM303 | G | KU131118 |  | KX503809 |
|  |  |  |  |  |  |  | HNSM304 | G | KU131118 |  | KU131185 |
|  |  |  |  |  |  |  | HNSM305 | G | KU131118 |  | KX503809 |
|  |  |  |  |  |  |  | HNSM306 | G | KU131118 |  |  |
|  |  |  |  |  |  |  | HNSM307 | G | KU131118 |  |  |
|  |  |  |  |  |  |  | HNSM308 | G | KU131118 |  |  |
|  |  |  |  |  |  |  | HNSM309 | G | KU131118 |  |  |
|  |  |  |  |  |  |  | HNSM310 | G | KU131118 |  |  |
|  |  |  |  |  |  |  | HNSM311 | G | KU131118 |  |  |
| **Wumuyu Cave, Yongding, Zhangjiajie, Hunan (18)** | Wulingshan M. | Laodongxi, Yuanjiang, Yangzte R. | 110.3E | 28.9N | O | 14 | HNWMY25 | B | KU131065 |  |  |
|  |  |  |  |  |  |  | HNWMY27 | B | KU131066 |  |  |
|  |  |  |  |  |  |  | HNWMY28 | G | KU131172 |  |  |
|  |  |  |  |  |  |  | HNWMY29 | B | KU131065 |  |  |
|  |  |  |  |  |  |  | HNWMY44 | B | KU131062 |  |  |
|  |  |  |  |  |  |  | HNWMY45 | B | KU131062 |  |  |
|  |  |  |  |  |  |  | HNWMY46 | B | KU131062 |  |  |
|  |  |  |  |  |  |  | HNWMY47 | B | KU131063 |  |  |
|  |  |  |  |  |  |  | HNWMY48 | B | KU131062 | KU131050 |  |
|  |  |  |  |  |  |  | HNWMY49 | B | KU131062 |  |  |
|  |  |  |  |  |  |  | HNWMY50 | B | KU131062 |  |  |
|  |  |  |  |  |  |  | HNWMY51 | B | KU131063 |  |  |
|  |  |  |  |  |  |  | HNWMY52 | B | KU131062 |  |  |
|  |  |  |  |  |  |  | HNWMY53 | B | KU131062 |  |  |
| **Yuanzi Cave, Shangdongjie, Sangzhi, Zhangjiajie, Hunan (19)** | Wulingshan M. | Lishui, Yangzte R. | 109.9E | 29.3N | O | 10 | HNSZSDJ78 | G | KU131131 |  |  |
|  |  |  |  |  |  |  | HNSZSDJ79 | G | KU131132 |  |  |
|  |  |  |  |  |  |  | HNSZSDJ80 | G | KU131133 |  |  |
|  |  |  |  |  |  |  | HNSZSDJ81 | G | KU131134 |  |  |
|  |  |  |  |  |  |  | HNSZSDJ82 | G | KU131135 | KU131061 |  |
|  |  |  |  |  |  |  | HNSZSDJ83 | G | KU131136 |  |  |
|  |  |  |  |  |  |  | HNSZSDJ84 | G | KU131137 |  |  |
|  |  |  |  |  |  |  | HNSZSDJ88 | G | KU131121 |  |  |
|  |  |  |  |  |  |  | HNSZSDJ91 | G | KU131121 |  |  |
|  |  |  |  |  |  |  | HNSZSDJ267 | G | KU131121 |  |  |
| **Maoping Cave, Gaoping, Longhui, Shaoyang, Hunan (20)** | Xuefeng M. | Zishui, Yangzte R. | 111.1E | 27.4N | O | 11 | HNLH723 | C | KU131082 |  |  |
|  |  |  |  |  |  |  | HNLH724 | C | KU131082 |  |  |
|  |  |  |  |  |  |  | HNLH725 | C | KU131082 |  |  |
|  |  |  |  |  |  |  | HNLH726 | C | KU131082 |  |  |
|  |  |  |  |  |  |  | HNLH734 | C | KU131082 |  |  |
|  |  |  |  |  |  |  | HNLH735 | C | KU131082 |  |  |
|  |  |  |  |  |  |  | HNLH737 | C | KU131082 |  |  |
|  |  |  |  |  |  |  | HNLH738 | C | KU131082 |  |  |
|  |  |  |  |  |  |  | HNLH740 | C | KU131082 |  |  |
|  |  |  |  |  |  |  | HNLH746 | C | KU131082 |  |  |
|  |  |  |  |  |  |  | HNLH749 | C | KU131082 |  |  |
| **Xinhuang, Huaihua, Hunan (21)** | Miaoling M., Yungui Plateau | Wushui, Yuanjiang, Yangzte R. | 109.4E | 27.3N | O | 1 | HNXH1008 | C |  |  |  |
| **Wufeng, Yichang, Hubei (22)** | Wulingshan M. | Qingjiang, Yangzte R. | 111.2E | 30.1N | O, S | 4 | HBWF524 | G | KU131117 |  |  |
|  |  |  |  |  |  |  | HBWF525 | G | KU131117 |  |  |
|  |  |  |  |  |  |  | HBWF527 | G | KU131117 |  |  |
|  |  |  |  |  |  |  | HBWF528 | G | KU131113 |  |  |
| **Jingan, Yichuan, Jiangxi (23)** | Molianjiu M. | Liaohe, Ganjiang, Yangzte R. | 115.1E | 28.9N | O | 10 | JXJA327 | D | KU131096 |  | KU131185 |
|  |  |  |  |  |  |  | JXJA328 | D | KU131097 |  | KU131185 |
|  |  |  |  |  |  |  | JXJA329 | D | KU131096 |  |  |
|  |  |  |  |  |  |  | JXJA330 | D | KU131096 |  |  |
|  |  |  |  |  |  |  | JXJA331 | D | KU131096 |  |  |
|  |  |  |  |  |  |  | JXJA332 | D | KU131090 |  | KU131185 |
|  |  |  |  |  |  |  | JXJA333 | D | KU131090 |  |  |
|  |  |  |  |  |  |  | JXJA334 | D | KU131090 |  |  |
|  |  |  |  |  |  |  | JXJA335 | D | KU131090 |  | KU131185 |
|  |  |  |  |  |  |  | JXJA336 | D | KU131090 | KU131044 |  |
| **Liukou, Xiuning, Huangshan, Anhui (24)** | Huangshan M. | Xinanjiang, Qiantangjiang R., Southeastern coastal river basin | 117.9E | 29.6N | O | 6 | AHHS692 | E | KU131107 |  | KU131185 |
|  |  |  |  |  |  |  | AHHS693 | E | KU131105 |  | KU131185 |
|  |  |  |  |  |  |  | AHHS694 | E | KU131106 |  |  |
|  |  |  |  |  |  |  | AHHS695 | E | KU131107 | KU131060 |  |
|  |  |  |  |  |  |  | AHHS696 | E | KU131107 |  |  |
|  |  |  |  |  |  |  | AHHS697 | E | KU131107 |  |  |
| **Xianliang Cave, Qingyuan, Lishui, Zhejiang (25)** | Donggongshan, Wuyishan M. | Xiaoxi, Oujiang R., Southeastern coastal river basin | 119.2E | 27.6N | O | 2 | ZJLSQY680 | E | KU131105 | KU131059 |  |
|  |  |  |  |  |  |  | ZJLSQY691 | E | KU131105 |  |  |
| **Huishui, Qiannan, Guizhou (26)** | Miaoling M., Yungui Plateau | Lianjiang, Hongshuihe, Xijiang, Pearl R. | 106.7E | 26.2N | O | 8 | GZHS575 | B | KU131070 |  |  |
|  |  |  |  |  |  |  | GZHS576 | B | KU131070 |  |  |
|  |  |  |  |  |  |  | GZHS577 | B | KU131070 |  | KU131185 |
|  |  |  |  |  |  |  | GZHS578 | B | KU131070 |  | KU131185 |
|  |  |  |  |  |  |  | GZHS579 | B | KU131070 |  | KU131185 |
|  |  |  |  |  |  |  | GZHS580 | B | KU131070 |  | KX503809 |
|  |  |  |  |  |  |  | GZHS581 | B | KU131070 |  | KU131187 |
|  |  |  |  |  |  |  | GZHS582 | B | KU131070 |  |  |
| **Longli Cave, Qiannan, Guizhou (27)** | Miaoling M., Yungui Plateau | Dushuihe, Wujiang, Yangzte R. | 106.9E | 26.5N | O | 2 | GZLL572 | B | KU131070 |  |  |
|  |  |  |  |  |  |  | GZLL573 | B | KU131070 |  |  |
| **Xiyejing Cave, Yanxia, Guiding, Qiannan, Guizhou (28)** | Miaoling M., Yungui Plateau | Hegouhe, Yuanjiang, Yangzte R. | 107.3E | 26.3N | O, S | 3 | GZGDYX583 | B | KU131070 | KU131054 |  |
|  |  |  |  |  |  |  | GZGDYX584 | B | KU131070 |  |  |
|  |  |  |  |  |  |  | GZGDYX585 | B | KU131070 |  |  |
| **Cengong, Qingdongnan, Guizhou (29)** | Miaoling M., Yungui Plateau | Wushui, Yuanjiang, Yangzte R. | 108.7E | 27.1N | O, S | 2 | GZCG233 | B | KU131071 |  |  |
|  |  |  |  |  |  |  | GZCG234 | B | KU131070 |  |  |
| **Ziyuan, Guilin, Guangxi (30)** | Maoershan, Nanling M. | Zishui, Yangzte R. | 110.6E | 26.0N | O, S | 17 | GXZY586 | C | KU131080 |  |  |
|  |  |  |  |  |  |  | GXZY587 | C | KU13108 | KU131055 |  |
|  |  |  |  |  |  |  | GXZY588 | C | KU131080 |  |  |
|  |  |  |  |  |  |  | GXZY589 | C | KU131080 |  |  |
|  |  |  |  |  |  |  | GXZY590 | C | KU131080 |  |  |
|  |  |  |  |  |  |  | GXZY591 | C | KU131080 |  |  |
|  |  |  |  |  |  |  | GXZY592 | C | KU131080 |  |  |
|  |  |  |  |  |  |  | GXZY593 | C | KU131080 |  |  |
|  |  |  |  |  |  |  | GXZY594 | C | KU131080 |  |  |
|  |  |  |  |  |  |  | GXZY595 | C | KU131080 |  |  |
|  |  |  |  |  |  |  | GXZY596 | C | KU131080 |  | KU131185 |
|  |  |  |  |  |  |  | GXZY597 | C | KU131081 |  | KU131185 |
|  |  |  |  |  |  |  | GXZY598 | C | KU131082 |  |  |
|  |  |  |  |  |  |  | GXZY599 | C | KU131082 |  | KU131185 |
|  |  |  |  |  |  |  | GXZY600 | C | KU131080 |  | KU131185 |
|  |  |  |  |  |  |  | GXZY601 | C | KU131080 |  | KU131185 |
|  |  |  |  |  |  |  | GXZY602 | C | KU131080 |  | KU131185 |
| **Xingan, Guilin, Guangxi (31)** | Maoershan, Yuechengling, Nanling M. | Lijiang, Xijiang, Pearl R. | 110.4E | 25.8N | O, S | 21 | GXXA603 | A | KU131174 |  | KX503810 |
|  |  |  |  |  |  |  | GXXA604 | A | KU131174 |  | KX503810 |
|  |  |  |  |  |  |  | GXXA605 | A | KU131174 |  | KX503810 |
|  |  |  |  |  |  |  | GXXA606 | A | KU131174 |  |  |
|  |  |  |  |  |  |  | GXXA607 | A | KU131174 |  |  |
|  |  |  |  |  |  |  | GXXA608 | A | KU131174 |  |  |
|  |  |  |  |  |  |  | GXXA609 | A | KU131174 | KU131056 |  |
|  |  |  |  |  |  |  | GXXA610 | A | KU131174 |  | KX503816 |
|  |  |  |  |  |  |  | GXXA611 | A | KU131175 |  | KU131185 |
|  |  |  |  |  |  |  | GXXA612 | A | KU131174 |  |  |
|  |  |  |  |  |  |  | GXXA613 | A | KU131176 |  | KX503810 |
|  |  |  |  |  |  |  | GXXA614 | A | KU131175 |  | KU131185 |
|  |  |  |  |  |  |  | GXXA615 | A | KU131175 |  |  |
|  |  |  |  |  |  |  | GXXA616 | A | KU131175 |  |  |
|  |  |  |  |  |  |  | GXXA617 | A | KU131175 |  |  |
|  |  |  |  |  |  |  | GXXA618 | A | KU131175 |  |  |
|  |  |  |  |  |  |  | GXXA619 | A | KU131177 |  | KU131185 |
|  |  |  |  |  |  |  | GXXA620 | A | KU131175 |  | KU131185 |
|  |  |  |  |  |  |  | GXXA621 | A | KU131175 |  | KX503810 |
|  |  |  |  |  |  |  | GXXA622 | A | KU131175 |  |  |
|  |  |  |  |  |  |  | GXXA623 | A | KU131178 |  | KU131185 |
| **Maoping, Jinggangshan, Jian, Jiangxi (32)** | Luoxiaoshan M. | Heshui, Ganjiang, Yangzte R. | 114.1E | 26.6N | O, S | 12 | JXJGS351 | D | KU131090 |  |  |
|  |  |  |  |  |  |  | JXJGS352 | D | KU131090 | KU131045 |  |
|  |  |  |  |  |  |  | JXJGS354 | D | KU131098 |  |  |
|  |  |  |  |  |  |  | JXJGS355 | D | KU131090 |  | KU131185 |
|  |  |  |  |  |  |  | JXJGS356 | D | KU131090 |  |  |
|  |  |  |  |  |  |  | JXJGS357 | D | KU131090 |  |  |
|  |  |  |  |  |  |  | JXJGS358 | D | KU131099 |  | KU131185 |
|  |  |  |  |  |  |  | JXJGS359 | D | KU131090 |  |  |
|  |  |  |  |  |  |  | JXJGS360 | D | KU131090 |  |  |
|  |  |  |  |  |  |  | JXJGS361 | D | KU131090 |  | KU131185 |
|  |  |  |  |  |  |  | JXJGS362 | D | KU131100 |  | KU131185 |
|  |  |  |  |  |  |  | JXJGS363 | D | KU131101 |  | KU131185 |
| **Lianzhou, Qingyuan, Guangdong (33)** | Mengzhuling, Nanling M. | Lianjiang, Beijiang, Pearl R. | 112.4E | 25.0N | O, S | 2 | GDLZ365 | D | KU131093 | KU131046 |  |
|  |  |  |  |  |  |  | GDLZ367 | D | KU131093 |  |  |

**Table S2**

| **Cave name** | **Locality (number)** | **Sample size** | **Sample stage** | **Sample site** | **Year of larvae captured** | **Year of tissues collected** |
| --- | --- | --- | --- | --- | --- | --- |
| Giant Salamander Hole^a^ | Daba, Xingwen, Yibin, Sichuan (12) | 17 | adult | In governmental rescue agencies | 2006 | 2016^c^ |
| Shuitianba Cave^b^ | Shuitianba, Longshan, Xiangxi, Hunan (15) | 20 | larva | In the field | 2015～2016 | 2015～2016 |
| Hekou Cave^a^ | Hekou, Sangzhi, Zhangjiajie, Hunan (16) | 10 | larva | In the field | 2015 | 2015 |
| Hupingshan Cave^b^ | Shimen, Changde, Hunan (17) | 21 | adult | In governmental rescue agencies | 2014～2015 | 2016^c^ |
| Wumuyu Cave^a^ | Wumuyu, Yongding, Zhangjiajie, Hunan (18) | 14 | larva | In the field | 2015 | 2015 |
| Yuanzi Cave^a^ | Shangdongjie, Sangzhi, Zhangjiajie, Hunan (19) | 10 | larva | In the field | 2013～2014 | 2013～2014 |
| Maoping Cave^a^ | Gaoping, Longhui, Shaoyang, Hunan (20) | 11 | adult | In governmental rescue agencies | 2006～2008 | 2015^c^ |
| Xianliang Cave^b^ | Qingyuan, Lishui, Zhejiang (25) | 1 | adult | In a local  resident’s aquarium | 2005 | 2015^c^ |
| Longli Cave^b^ | Longli, Qiannan, Guizhou (27) | 2 | adult | Small farm | 2017 | 2018^c^ |
| Xiyejing Cave^a^ | Yanxia, Guiding, Qiannan, Guizhou (28) | 3 | adult | In governmental rescue agencies | 2009 | 2014^c^ |
| Total size or year |  | 109 |  |  | 2005～2017 | 2013～2018 |

**Table S3**

| **ID** | **Primer** | **Sequence (5'-3')** |
| --- | --- | --- |
| 1 | L17 | TAAAGCGTGGCACTGAAAATGC |
|  | H947 | GTCGTAACATGGTAAGTGTACCG |
| 2 | L747 | AATGGGAAGAAATTGGCTAC |
|  | H1905 | TTTGGTAAACAGGCGAAGTTC |
| 3 | L1758 | GATATTTAACGAACCATATTGAAGG |
|  | H2799 | GCAACAGCTAGTAAGATTGG |
| 4 | L2621 | CCTAAATAAGGGCTAGCTAAG |
|  | H3675 | TAGGAGGTACGCCTATTCAG |
| 5 | L3567 | CGCTATGATCAACTAATACACC |
|  | H4566 | ATGGGCTGATAGGATTAGTAGTG |
| 6 | L4435 | TCATCAATTGCCCATTTAGGTTG |
|  | H5497 | TCACAAAGGCATGGGCTGTTAC |
| 7 | L5351 | GCACCCTATATTTAGTATTCGGTG |
|  | H6471 | GATGGCAAATACAGCTCCTATTG |
| 8 | L6296 | CTATTAAATGAGACGCCGCAATAC |
|  | H7174 | CTTGGGCATCTATGGTATTAGTG |
| 9 | L7074 | TCATGATCATGCACTTATAGCTG |
|  | H8065 | GTACAAGTCAGATTTGAAGGGT |
| 10 | L7911 | CCACAATCTTGAAACTGACCATG |
|  | H8961 | GGGTAATTCCAATAGGAGGTCA |
| 11 | L8790 | ACATTTCAAGGCCATCATACAACC |
|  | H9693 | ATTGTGATGCTCATGGTGTTGGT |
| 12 | L9558 | CCATATGAGTGCGGGTTTGATCCT |
|  | H10545 | ATGTAGGGATCAAAGTGGTCTCAA |
| 13 | L10416 | ACCACACTAAGTTGACAACGCAC |
|  | H11469 | GAACAATCAGTAAATGTTCTCGGGT |
| 14 | L11345 | TGGCATTAGCACCGTAATTACAGC |
|  | H12463 | GGACCTTCTATAGCAGCTGGTAATC |
| 15 | L12290 | CCTAATTATTACAATTGCCTGATTTGC |
|  | H13255 | AGGTCTACTGCTAGGGTTAGACCTAC |
| 16 | L13127 | CATTAAACGATTAGCCATAGGCAGT |
|  | H13960 | TGGCTTCTATTTGTGGGTGTTCTGT |
| 17 | L13828 | TATGGTTCCGCAGCCAAAGAAGCAG |
|  | H14986 | GATTGATCGGAGAATTGCATAGGCAA |
| 18 | L14764 | ATGAAACAGGGTCAAGCAATCCAAC |
|  | H16062 | AGATGAGGGCAGACTCAGTTATG |
| 19 | L15890 | AGATCTATGGACACTTCTAGTAGAG |
|  | H191 | TGTGGTGCCTGATATCTGCTC |
| 20 | RAG2_F | GTCATGTCTGCCGTGTATC |
|  | RAG2_R | TCTCCCTCATCGTCCTCGTTGT |

**Table S4**

| Scheme Number | Composition | Best model |
| --- | --- | --- |
| 1 | ATP6,ATP8,CYTB,ND1,ND2,ND3,ND4,ND4L,ND5 | GTR+I+G |
| 2 | COI,COII,COIII, tRNA | GTR+I+G |
| 3 | ND6 | GTR+G |
| 4 | 2rRNAs | GTR+I+G |

**Table S5**

|  | n*/*N | *h* | ɵ_π (%)_ | ɵ*_w_* _(%)_ |
| --- | --- | --- | --- | --- |
| 1 | 4/6 | 0.800±0.172 | **1.046** | **1.317** |
| 2 | 1/2 | - | **-** | **-** |
| 3 | 5/23 | 0.087±0.078 | **0.008** | **0.025** |
| 4 | 1/9 | 0 | 0 | 0 |
| 5 | 6/14 | 0.275±0.148 | **0.027** | **0.059** |
| 6 | 2/17 | 0 | 0 | 0 |
| 7 | 2/7 | 0.286±0.196 | **0.27** | **0.38** |
| 8 | 1/1 | - | - |  |
| 9 | 3/6 | 0.733±0.155 | **0.357** | **0.370** |
| 10 | 6/11 | 0.818±0.083 | 0.352 | 0.289 |
| 11 | 2/10 | 0.200±0.154 | **0.019** | **0.033** |
| 12 | 4/17 | 0.221±0.121 | **0.104** | **0.139** |
| 13 | 3/13 | 0.295±0.156 | **0.671** | **1.268** |
| 14 | 3/8 | 0.679±0.122 | **1.040** | **1.400** |
| 15 | 2/20 | 0 | 0 | 0 |
| 16 | 6/10 | 0.711±0.117 | 1.544 | 1.210 |
| 17 | 3/21 | 0.095±0.084 | **0.009** | **0.026** |
| 18 | 5/14 | 0.473±0.136 | **0. 681** | **1.235** |
| 19 | 8/10 | 0.933±0.077 | 0.517 | 0.398 |
| 20 | 1/11 | 0 | 0 | 0 |
| 21 | 1/1 | - | - | - |
| 22 | 2/4 | - | - | - |
| 23 | 3/10 | 0.644±0.101 | 0.072 | 0.067 |
| 24 | 3/6 | 0.600±0.215 | 0.082 | 0.083 |
| 25 | 1/2 | - | - | - |
| 26 | 1/8 | 0 | 0 | 0 |
| 27 | 1/2 | - | - | - |
| 28 | 1/3 | - | - | - |
| 29 | 2/2 | - | - | - |
| 30 | 1/17 | 0 | 0 | 0 |
| 31 | 5/21 | 0.571±0.052 | 0.209 | 0.128 |
| 32 | 4/12 | 0.455±0.170 | **0.110** | **0.220** |
| 33 | 1/2 | - | - | - |

**Table S6**

|  | A | B | C | D | E | F |
| --- | --- | --- | --- | --- | --- | --- |
| A |  |  |  |  |  |  |
| B | 3.47 |  |  |  |  |  |
| C | 3.43 | 2.57 |  |  |  |  |
| D | 3.59 | 2.55 | 1.92 |  |  |  |
| E | 4.32 | 3.22 | 2.83 | 2.68 |  |  |
| F | 4.23 | 3.10 | 3.19 | 3.19 | 3.78 |  |
| G | 4.37 | 3.96 | 3.63 | 3.12 | 3.99 | 2.67 |

**Figure S1**

**
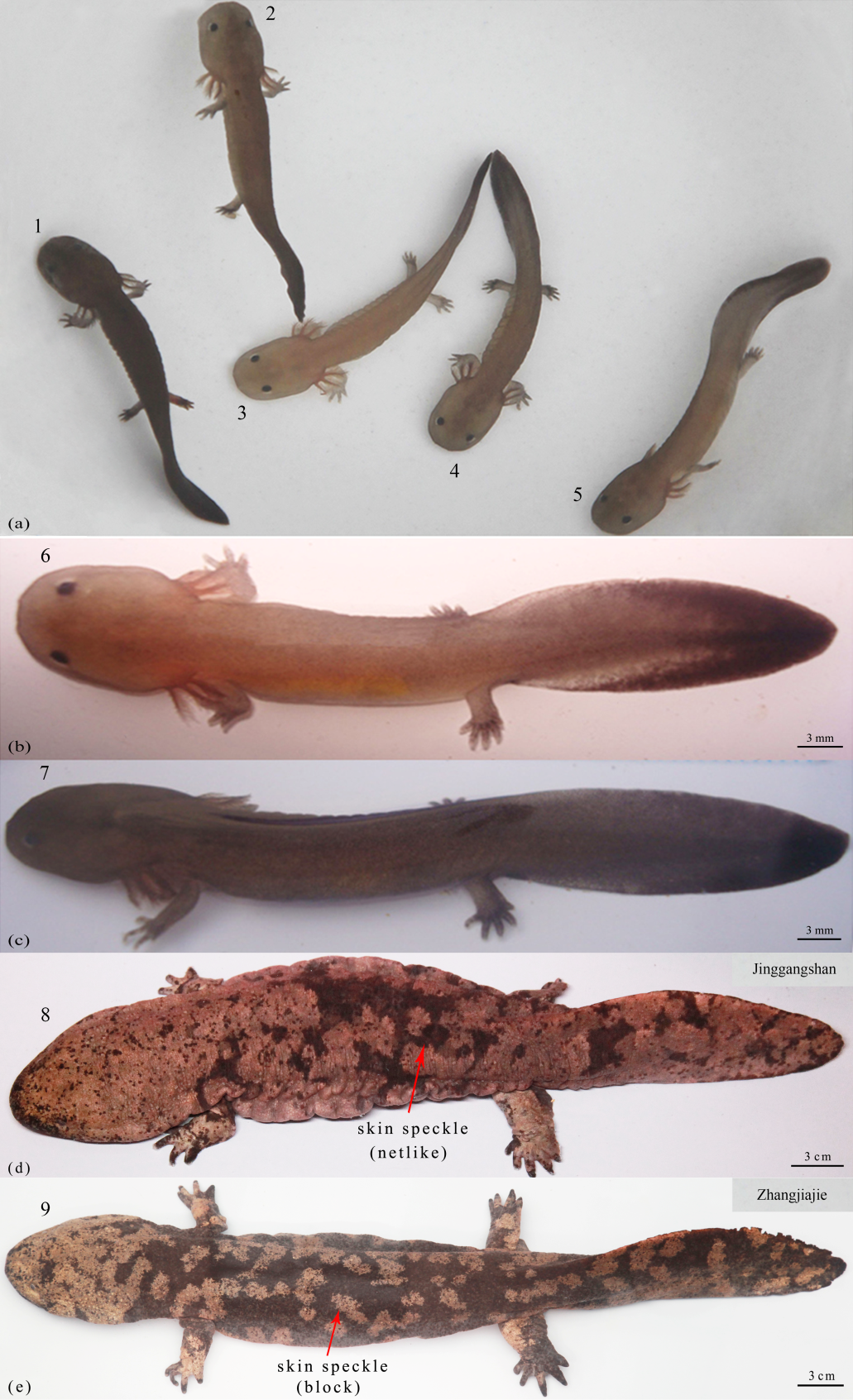
**

**Figure S2**

**
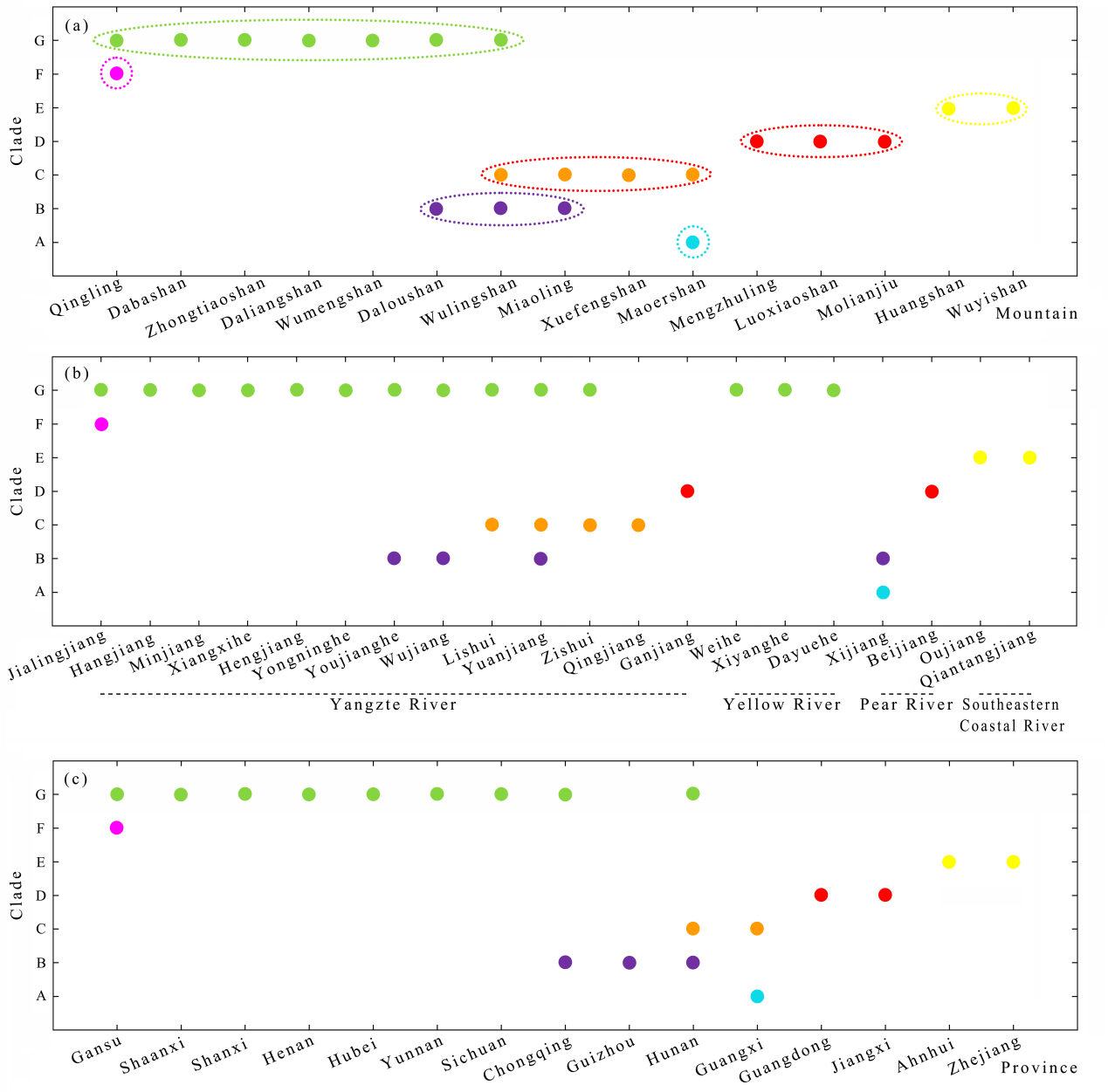
**

**Figure S3**

**
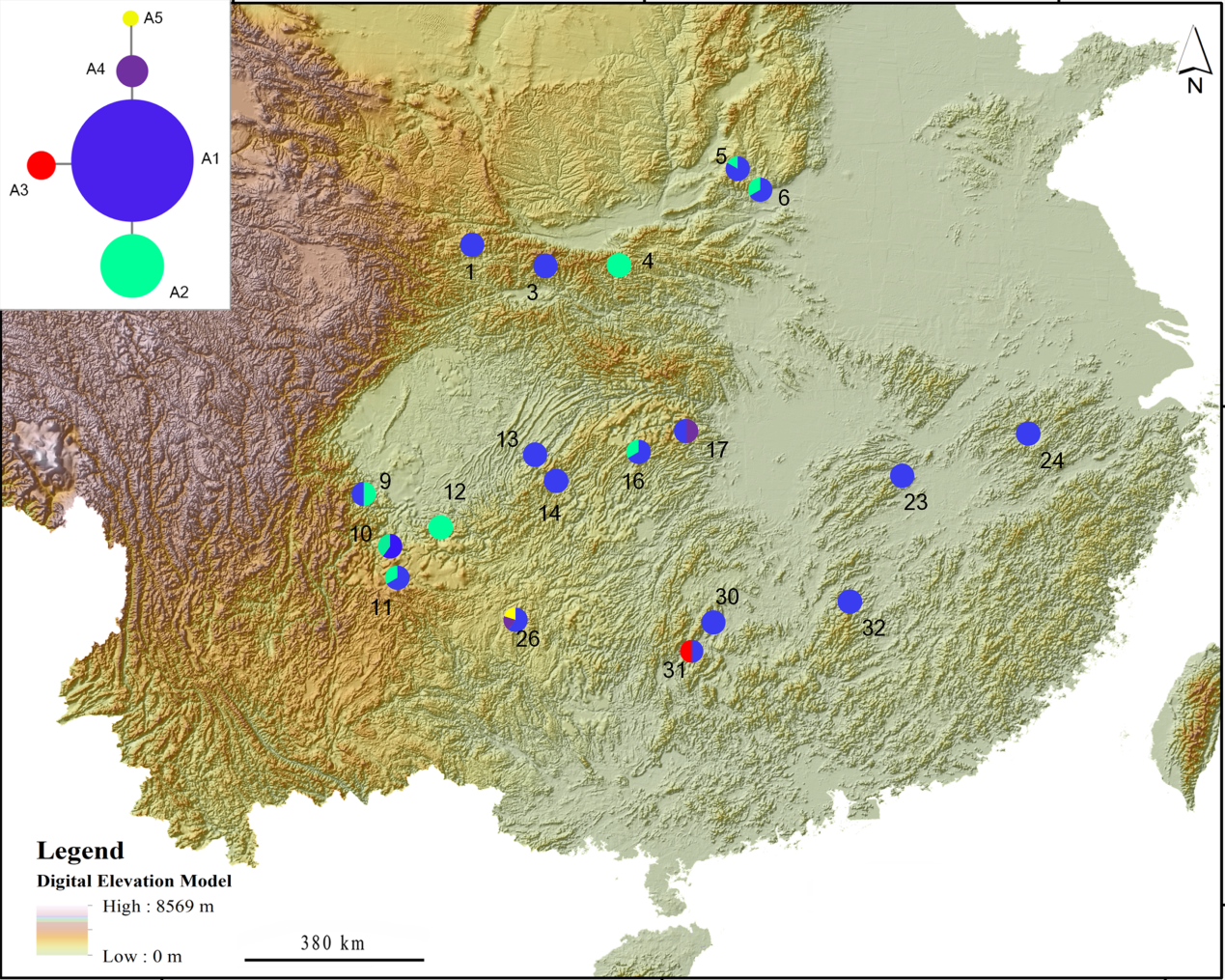
**

**Figure S4**

**
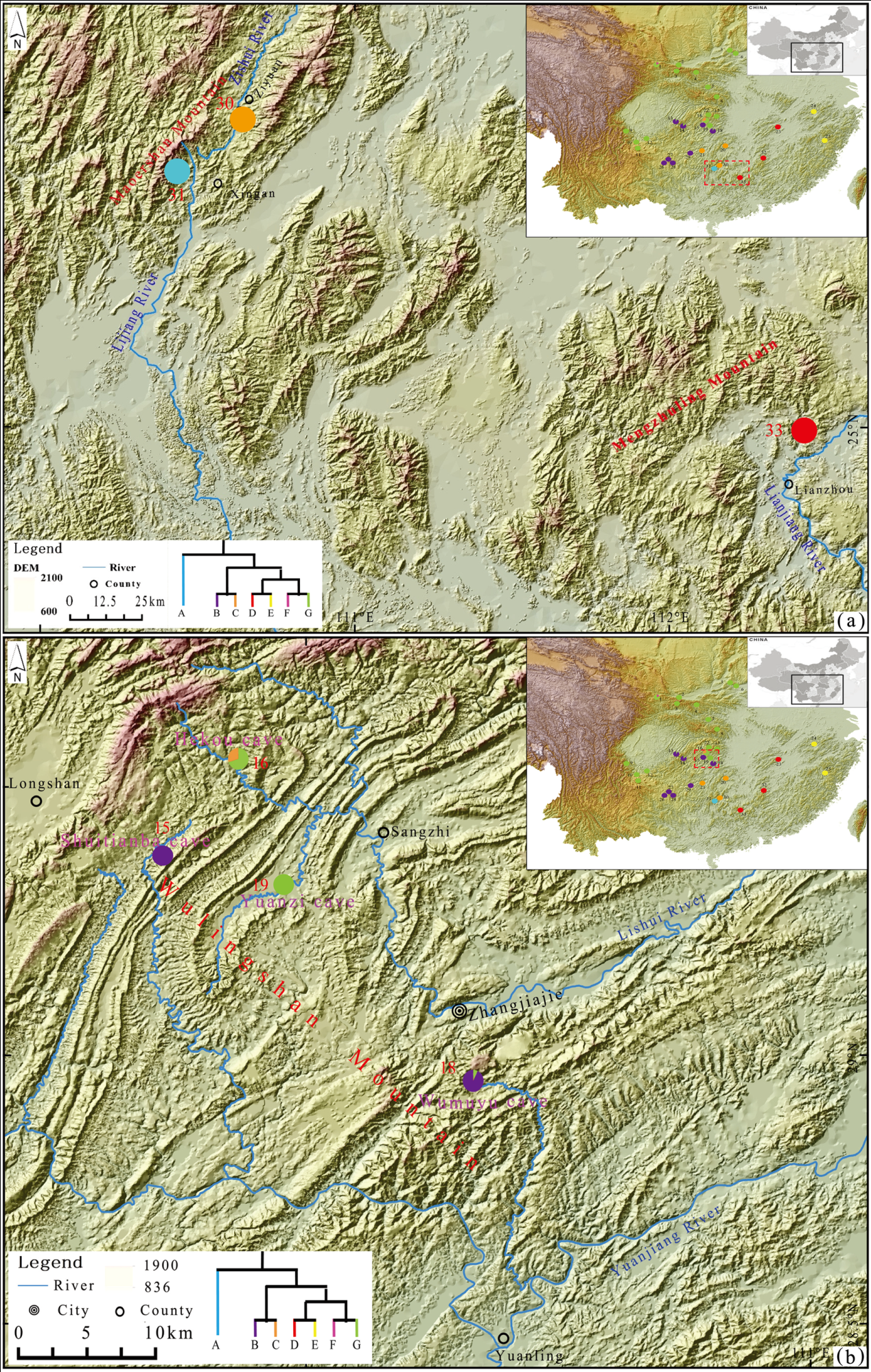
**
